# Molecular Targets in Modern Cosmetic Science

**DOI:** 10.3390/molecules31173036

**Published:** 2026-08-28

**Authors:** Justyna Popiół-Adamska, Karolina Łagosz, Michał Kolisz, Klaudia Uniwersał, Małgorzata Kabat-Walewska, Yelyzaveta Shuha, Nattaya Lourith, Mayuree Kanlayavattanakul, Agnieszka Gunia-Krzyżak

**Affiliations:** 1 Department of Pharmaceutical Biochemistry, Faculty of Pharmacy, Jagiellonian University Medical College, 30-688 Krakow, Poland; justyna.popiol@uj.edu.pl; 2 Department of Experimental Dermatology and Cosmetology, Faculty of Pharmacy, Jagiellonian University Medical College, 30-688 Krakow, Poland; 3 Doctoral School of Medical and Health Sciences, Jagiellonian University Medical College, 30-688 Krakow, Poland; 4 School of Cosmetic Science, Mae Fah Luang University, Chiang Rai 57100, Thailand; nattayal@mfu.ac.th (N.L.); mayuree@mfu.ac.th (M.K.); 5 Phytocosmetics and Cosmeceuticals Research Group, School of Cosmetic Science, Mae Fah Luang University, Chiang Rai 57100, Thailand

**Keywords:** telomerase, sirtuins, tyrosinase, matrix metalloproteinases, melanocortin receptors, CD44, anti-aging, pigmentation, cosmetic science, molecular targets, nail care, hair care

## Abstract

The field of cosmetic science is rapidly evolving due to advances in skin biology research and increasing demand for evidence-based, personalized skincare solutions. A key aspect of this progress is the targeted modulation of biological macromolecules involved in skin aging, pigmentation, inflammation, and tissue regeneration. This review highlights major molecular targets in modern cosmetic research, focusing on telomerase, histone deacetylases (particularly sirtuins), matrix metalloproteinases, antioxidant enzymes, CD44 receptor, tyrosinase, microphthalmia-associated transcription factor, and melanocortin receptors, all of which regulate processes such as DNA repair, gene expression, extracellular matrix remodeling, and melanogenesis. Recent findings indicate that natural and synthetic compounds can effectively modulate these targets, supporting anti-aging and depigmenting strategies. Telomerase activators and sirtuin modulators show potential in maintaining cellular homeostasis and delaying skin aging. In pigmentation research, the transition from mushroom to human tyrosinase assays has enabled development of more selective inhibitors, including thiamidol. The review also addresses molecular targets relevant to inflammatory skin disorders, scars, and skin atrophy. Advances in molecular and biotechnological methods are expected to improve mechanistic understanding and support the rational design of safer, more effective next-generation cosmetic ingredients and formulations.

## 1. Introduction

Since ancient times, humans have sought to enhance their appearance using various substances to beautify, protect, or modify the skin and hair. Across different cultures and historical periods, external appearance has been closely associated with health, youthfulness, social status, and personal identity, values that remain highly relevant until today. Traditionally, cosmetic practices relied heavily on natural ingredients such as plant extracts, oils, and clays, which were used empirically, often without understanding the mechanisms underlying their effects [1,2].

In recent years, the field of cosmetic science has undergone a profound transformation, evolving from traditional formulations to a science-based discipline that integrates dermatological, biochemical, and molecular knowledge. Advances in biology, biotechnology, and material sciences have enabled not only the identification and development of novel cosmetic ingredients, either synthetic or derived through advanced biotechnological processes, but also the discovery of specific molecular targets, particularly biological macromolecules, that these ingredients can selectively modulate to influence key skin-related pathways such as aging, pigmentation, inflammation, or regeneration [3,4].

A notable manifestation of this shift is the emerging concept of longevity cosmeceuticals, which represents a new generation of cosmetic actives designed to directly target the hallmarks of skin aging, extend skin healthspan (“skinspan”), and be validated through biomarker-based efficacy testing in clinical settings. Unlike conventional anti-aging products aimed primarily at reducing visible signs of aging, these compounds operate at the molecular level, integrating cosmetic application with geroscience and preventive dermatology [5].

The global cosmetics market was estimated at 295.95 billion USD in 2023 and is projected to reach 445.98 billion USD by 2030. In 2023, skincare products accounted for the largest segment, with a 43.3% revenue share, followed by hair care, makeup, fragrance, and others. This dynamic growth is driven by increasing consumer awareness of personal appearance, the rising demand for evidence-based and personalized skincare, and a preference for natural, non-toxic, and sustainable ingredients. Importantly, cosmetic users now expect scientifically proven effectiveness, ideally demonstrated under well-designed, animal-free experimental conditions [6,7].

Irrespective of their origin or technological complexity, cosmetic products remain subject to specific regulatory definitions that distinguish them from medicinal products and medical devices. According to Regulation (EC) No. 1223/2009 [8], cosmetic products are intended to be placed in contact with the external parts of the human body, including the epidermis, hair system, nails, lips, and external genital organs, or with the teeth and the mucous membranes of the oral cavity. Comparable regulatory definitions are also established in other major jurisdictions, including the United States under the Federal Food, Drug, and Cosmetic Act and the ASEAN Cosmetic Directive which harmonizes cosmetic regulations across the member states of the Association of Southeast Asian Nations [9,10]. Thus, regardless of the sophistication of their active ingredients, including peptides, biotechnology-derived molecules, or extracellular vesicles, cosmetic products are applied to the body through cosmetic routes consistent with these regulatory definitions, predominantly topical application. In the European Union, they are also distinguished from medicinal products by the absence of a principal pharmacological, immunological, or metabolic mode of action [8]. However, modern cosmetic science increasingly views active ingredients as biofunctional agents that can interact with specific molecular pathways in the skin. One of the key strategies in the search for next-generation cosmetic actives is the design and selection of molecules that precisely modulate the activity of biological macromolecules such as enzymes, receptors, and structural proteins.

This review focuses on selected biological macromolecules as molecular targets for cosmetic intervention, whose modulation has shown promise in skin-related processes including aging, pigmentation, inflammation, and regeneration. The manuscript presents recent findings regarding telomerase, histone deacetylases (notably sirtuins), matrix metalloproteinases, tyrosinase, and melanocortin receptors, among others. The article also highlights molecular targets relevant to (photo)anti-aging strategies, skin atrophy, melanogenesis inhibition and sunless tanning, as well as inflammatory skin disorders, particularly atopic dermatitis. Moreover molecular targets in hair and nail care were also discussed. By outlining current research on these macromolecular targets, this review aims to provide a comprehensive framework for the rational development of innovative and effective cosmetic formulations.

The review is organized as follows: molecular targets in (photo)anti-aging strategies are addressed first, covering telomerase, histone deacetylases, matrix metalloproteinases, and antioxidant enzymes, followed by the CD44 receptor in the context of skin atrophy. Subsequent sections address pigmentation regulation targets (tyrosinase, MITF, cAMP signaling, melanocortin receptors), molecular targets in scar formation, and finally targets relevant to atopic dermatitis as molecular targets in hair and nail care.

## 2. Molecular Targets in Modern (Photo)Anti-Aging Strategies

Aging is a multifactorial biological process driven by the progressive decline of cellular and tissue homeostasis. Skin aging is slow and is monitored by internal factors, including heredity and cellular metabolism, and by external factors, including UV exposure, environmental pollution, and oxidative stress. Of these, chronic UV exposure is especially deleterious because it induces skin aging through a process of photoaging [11,12]. At the molecular level, several connected biochemical pathways contribute to skin aging. Oxidative stress, resulting from the accumulation of reactive oxygen species (ROS), damages DNA, proteins, and lipids, leading to functional decline and cellular senescence. Telomere shortening and impaired DNA repair mechanisms exacerbate genomic instability, while mitochondrial dysfunction reduces energy production and increases ROS generation, creating a vicious cycle of cellular damage. Nutrient-sensing pathways, including insulin/IGF-1 signaling and mTOR (mechanistic target of rapamycin), play pivotal roles in regulating growth and metabolism; their dysregulation is strongly associated with age-related decline in skin structure and function [13,14,15].

In the context of skin aging, these molecular events manifest as reduced collagen and elastin synthesis, extracellular matrix remodeling, epidermal thinning, impaired barrier function, and possible chronic low-grade inflammation. Advanced glycation end products (AGEs) accumulate over time, crosslinking structural proteins and further compromising skin elasticity. Matrix metalloproteinases (MMPs), activated by both intrinsic aging and external factors such as UV exposure, degrade collagen and elastin fibers, accelerating visible signs of aging [13,16].

Biomarkers provide measurable indicators of these molecular changes and serve as a critical bridge connecting mechanistic understanding of skin aging with the evaluation of targeted intervention strategies. By quantifying key molecular events, they allow researchers to assess whether a cosmetic ingredient meaningfully modulates a relevant pathway. Primary biomarkers include telomere length, ROS levels, MMP activity, and sirtuin activity, which collectively reflect cellular senescence, oxidative stress, and epigenetic regulation. Secondary markers, such as pro-inflammatory cytokines (IL-6, TNF-α) and AGEs, indicate cumulative molecular damage and tissue-level deterioration [5,13]. Epigenetic clocks (biomarkers of biological aging) are particularly important, as they capture the influence of genetics, lifestyle, and environmental factors on the aging process. These biomarkers can be used to predict disease risk and to monitor changes occurring in the body over time. Biomarker development has been greatly accelerated by modern technologies. Omics approaches, such as genomics, proteomics, and metabolomics, enable the analysis of thousands of molecules simultaneously, rather than focusing on single indicators. In addition, machine learning and deep neural networks allow for rapid identification of meaningful patterns in large and complex biological datasets, improving the precision of biological age estimation. Importantly, biomarkers are not fixed and can be influenced by lifestyle factors such as diet and physical activity [17,18].

In response to these challenges, current anti-aging strategies are becoming more and more oriented towards those molecular pathways, as opposed to merely treating observable manifestations. An emerging approach, referred as “genocosmetics” or genomics-based cosmetics, involves innovative cosmetic products enriched with bioactive ingredients capable of modulating gene regulatory pathways [19]. These products may be working on several levels: they might help to stabilize telomeres, but also modulate epigenetic regulators such as microRNAs and histone-modifying enzymes, enhance cellular metabolism, and reduce oxidative stress [20,21]. Epigenetic strategies are focused on balanced up- and downregulation of genes related to aging, mostly by the regulation of enzymes that modify histones or through microRNA activity in the cells. Naturally occurring epigenetic mechanisms including DNA methylation, histone acetylation, or microRNA regulation affect skin aging by controlling processes like repair of DNA, regeneration of cells, production of collagen, and dealing with oxidative damage. Also matrix metalloproteinases have emerged as promising molecular targets for anti-aging formulations [12,22,23].

### 2.1. Telomerase

Telomeres are specialized structures composed of repetitive DNA sequences. They are located at the termini of eukaryotic chromosomes and protect against chromosomal damage and fusion. Telomerase is an enzyme that counters telomere shortening. Telomeres shorten naturally with each cell division and play an important role in cellular aging, including that of the skin. Telomere shortening was associated with reduced regenerative ability, and loss of tissue function. In the skin, in which rapid renewal is required to protect the body from external assaults, reduced telomerase activity slows down the functions of regeneration, resulting in the rapid appearance of skin aging, translated in the form of wrinkles, reduced elasticity, and dryness [23,24,25].

Telomerase activators seek to prolong the life of a cell by preventing or even reversing telomere shortening. In fact, it is documented that by stimulating telomerase activity, fibroblasts can proliferate and produce collagen more effectively, leading to better skin quality [26]. In cosmetic science, bioactive molecules like some polyphenols, peptides, and plant extracts could possibly be used to regulate telomerase activity and promote regeneration; however, this is mostly due to their antioxidant effects [27,28]. Few in vitro and/or in vivo studies showed telomerase activation by certain compounds or formulations. Tests performed in human peripheral blood mononuclear cells proved this activity for *Centella asiatica* extract formula, oleanolic acid (Table 1), and maslinic acid [29]. Cycloastragenol, a triterpenoid saponin derived from *Astragalus membranaceus* showed antioxidant, anti-inflammatory, anti-aging, anti-apoptotic, and cardiovascular protective effects, telomerase activation and lengthening of telomeres was proved in mice after oral supplementation and in various cell lines such as fibroblasts, and keratinocytes [30]. Epitalon, a tetrapeptide (Ala-Glu-Asp-Gly), among its broad beneficial activity spectrum, was shown to induce the expression of telomerase, telomerase activity, and telomere elongation in various human cells [31].

However, increased or dysregulated telomerase activity may theoretically raise safety concerns, as sustained telomere maintenance can support prolonged cellular proliferation and is associated with the biology of many cancers [32,33]. In the context of cosmetic applications, this risk is considered largely theoretical, given the generally limited penetration and biological activity of cosmetic ingredients and the absence of evidence demonstrating that topical cosmetic products can induce clinically relevant telomerase activation. Nevertheless, the long-term effects of ingredients proposed to modulate telomerase activity warrant further investigation.

### 2.2. Histone Deacetylases

Histone deacetylases (HDACs) are enzymes responsible for deacetylation of histones, which act as epigenetic factors regulating DNA transcription [34]. Among HDACs, the sirtuin family, which are nicotinamide adenine dinucleotide (NAD+) dependent enzymes, has generated particular attention as a molecular target for anti-aging cosmetic ingredients due to their participation in chromatin stability, homeostasis, and aging [35]. However, the sirtuins are both deacetylases and ADP-ribosyltransferases, and their substrates include also nonhistone proteins, so the biological effects of their modulators may be unpredictable [36]. NAD+ itself, as a fundamental regulator of the activities of sirtuins on histone deacetylation, was proved to restore cellular dysfunctional bioenergetics and delay premature senescence in UV-irradiated skin, as well as maintain stratum corneum activity affecting aging of cutaneous tissues [35,37,38].

HDACs inhibitors, including sirtuin inhibitors, are considered novel promising anti-aging cosmetic active ingredients. Tests performed in several animal models proved their ability to increase the lifespan. Acetylation of histones located near pro-longevity genes causes an increase in their transcription and activation of the cellular stress response. Among multiple beneficial activities of HDACs inhibitors, promotion of stem cell stemness, improvement of mitochondrial function and biogenesis, amelioration of nutrient signaling, reduction in abnormal protein aggregation, increasing of genomic stability, elongation of telomeres, and promotion of epigenetic maintenance should be mentioned [39]. Based on the fact that the acetylation of histones regulates transcription, cell proliferation and differentiation, HDAC inhibitors have been tested so far in clinical trials as promising drugs for cancer therapy and received approval for the treatment of cutaneous T-cell lymphoma [34,40]. However, the translation of HDAC modulators from pharmacological to cosmetic applications requires careful consideration of concentration, safety profiles, and long-term epidermal effects, necessitating specific proof of efficacy under cosmetic use conditions. However, sirtuin modulators are mostly considered as anti-aging agents [39]. Several sirtuin activators have been identified, including butein, myricetin, quercetin, and resveratrol (Table 1), while sirtuin inhibition properties were proved among others for nicotinamide (Table 1) and cambinol [36].

**Table 1:** Representative cosmetic active ingredients targeting biological macromolecules and used in anti-aging formulations.

Cosmetic Ingredient	Biological Action
Nicotinamide (Niacinamide) 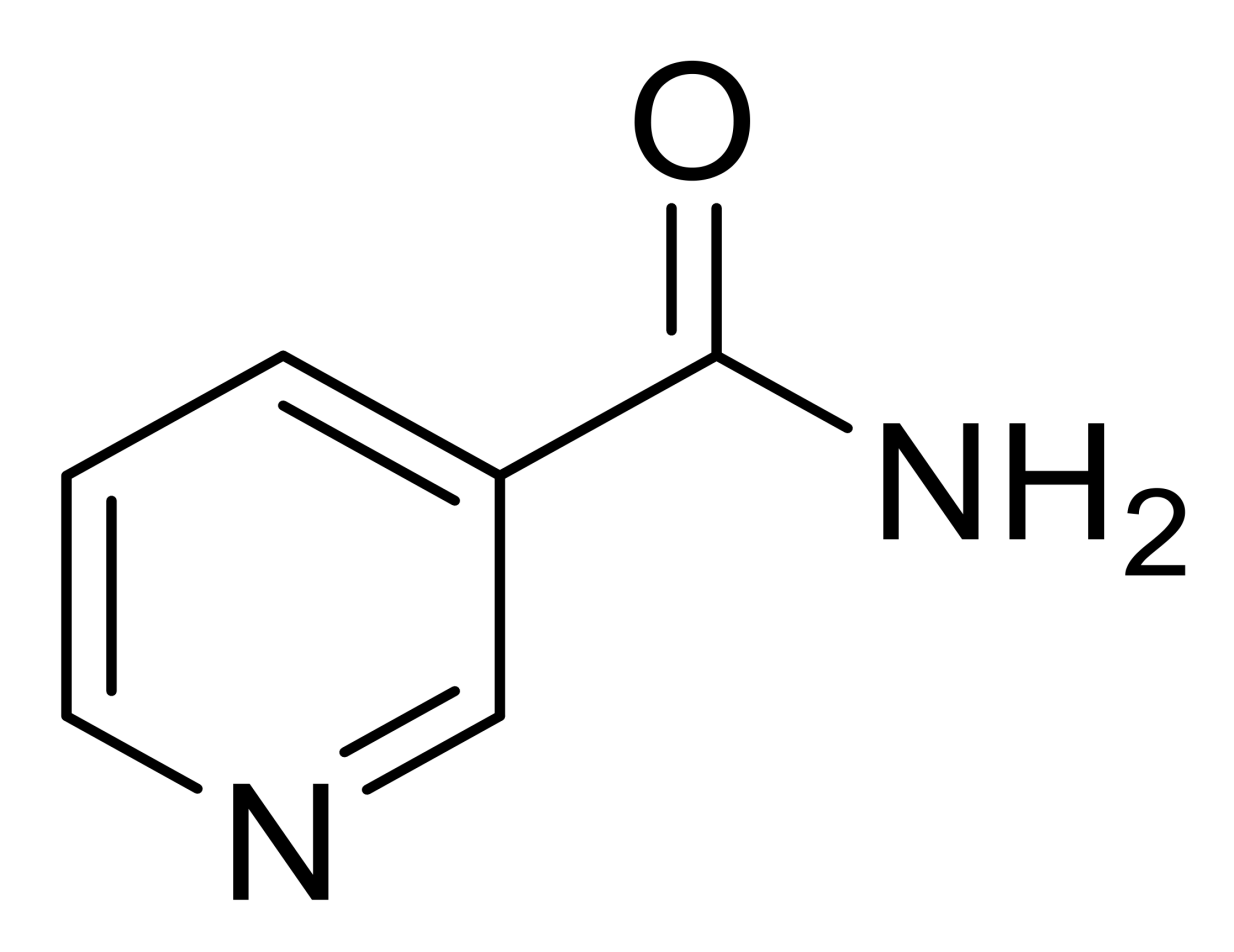	Inhibition of sirtuin; Inhibition of melanosome transfer from melanocytes to keratinocytes—improving skin pigmentation; Inhibition of ROS production in cells, attenuation of the production of pro-inflammatory cytokines IL-6 and IL-8; Extending the lifespan of human fibroblasts, possibly through reduction in mitochondrial activity and ROS production; Stabilizing effect on epidermal barrier function after topical application; Stimulating effect on ceramide synthesis; Improving the visible signs of aging [36,41,42,43,44,45,46,47]
Glycyl-L-histidyl-L-lysine-copper; (INCI: Copper Tripeptide-1, GHK-Cu) 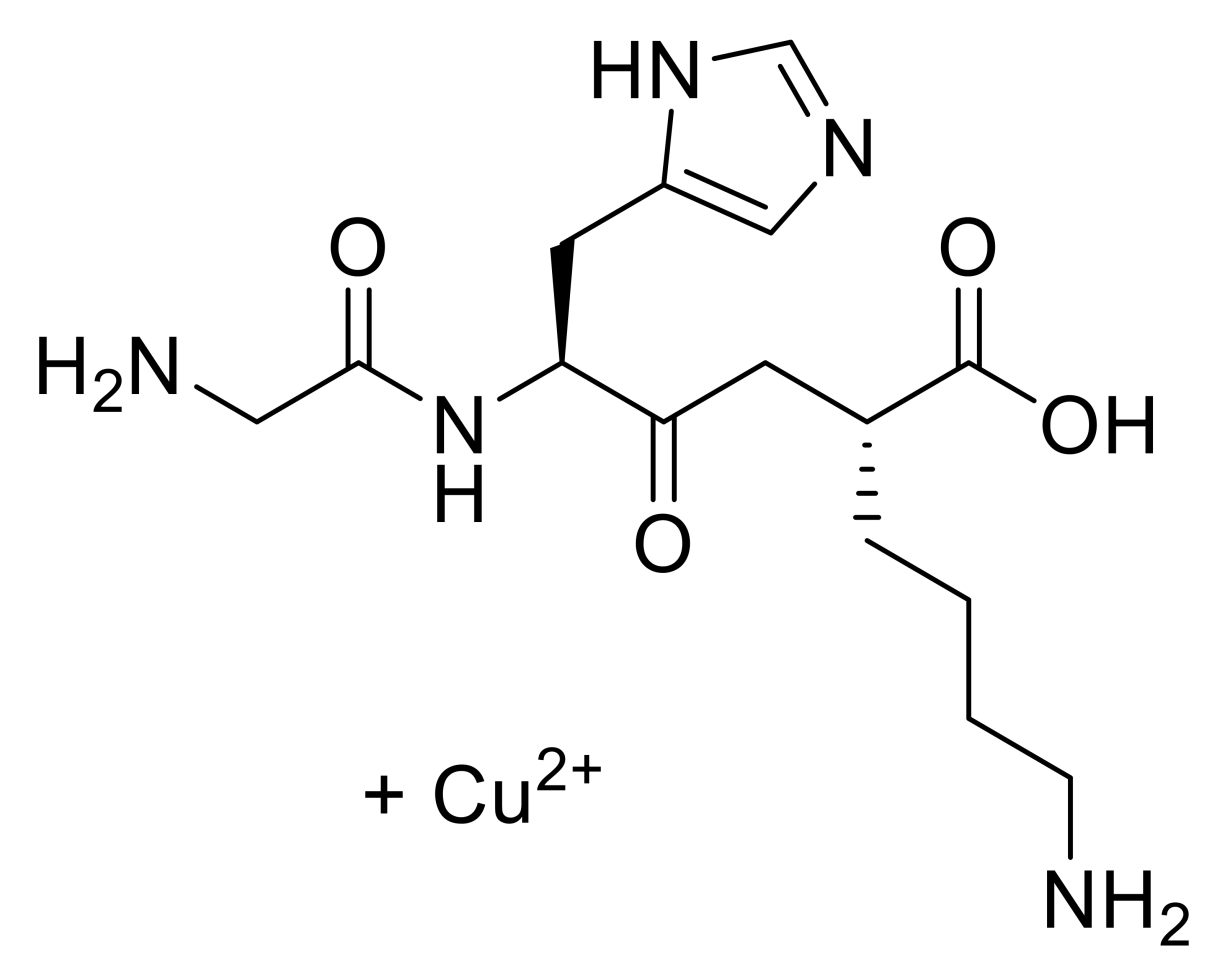	Stimulation of collagen, elastin, and glycosaminoglycan synthesis, reduction in wrinkle volume; Suppression of collagen-degrading proteolytic activity [48,49,50]
Resveratrol (3,5,4′-trans-trihydroxystilbene) 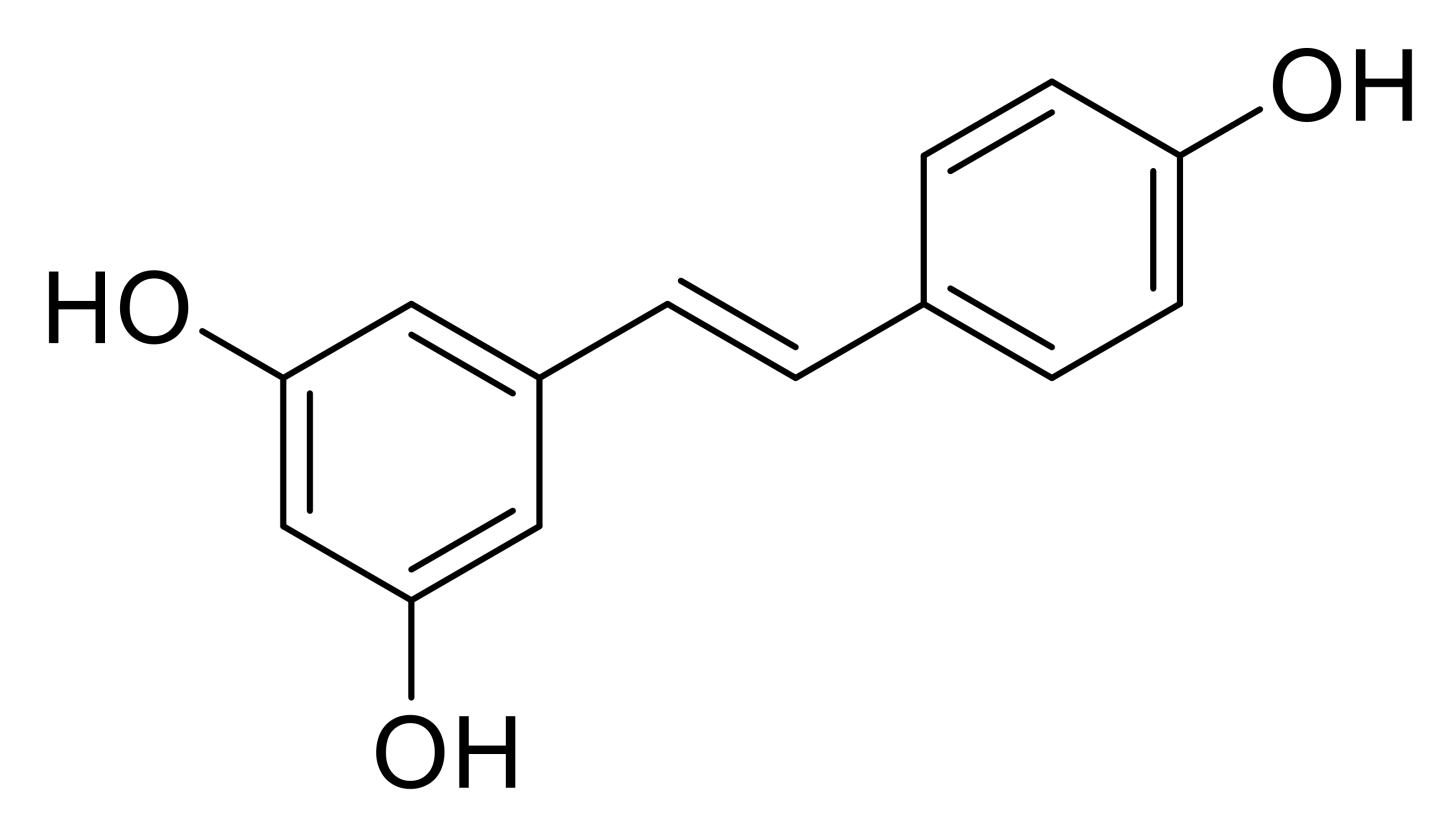	Inhibition of ROS production triggered by nitric oxide; Protection from apoptosis and MMPs damage caused by sodium nitroprusside in HaCaT cell line; Suppression of MMP-1 release, activation of antioxidant Nrf2 pathway, anti-inflammatory activity; Inhibition of tyrosinase [36,51,52,53,54]
γ-Mangostin (1,3,6,7-tetrahydroxy-2,8-bis(3-methylbut-2-en-1-yl)-9*H*-xanthen-9-one) 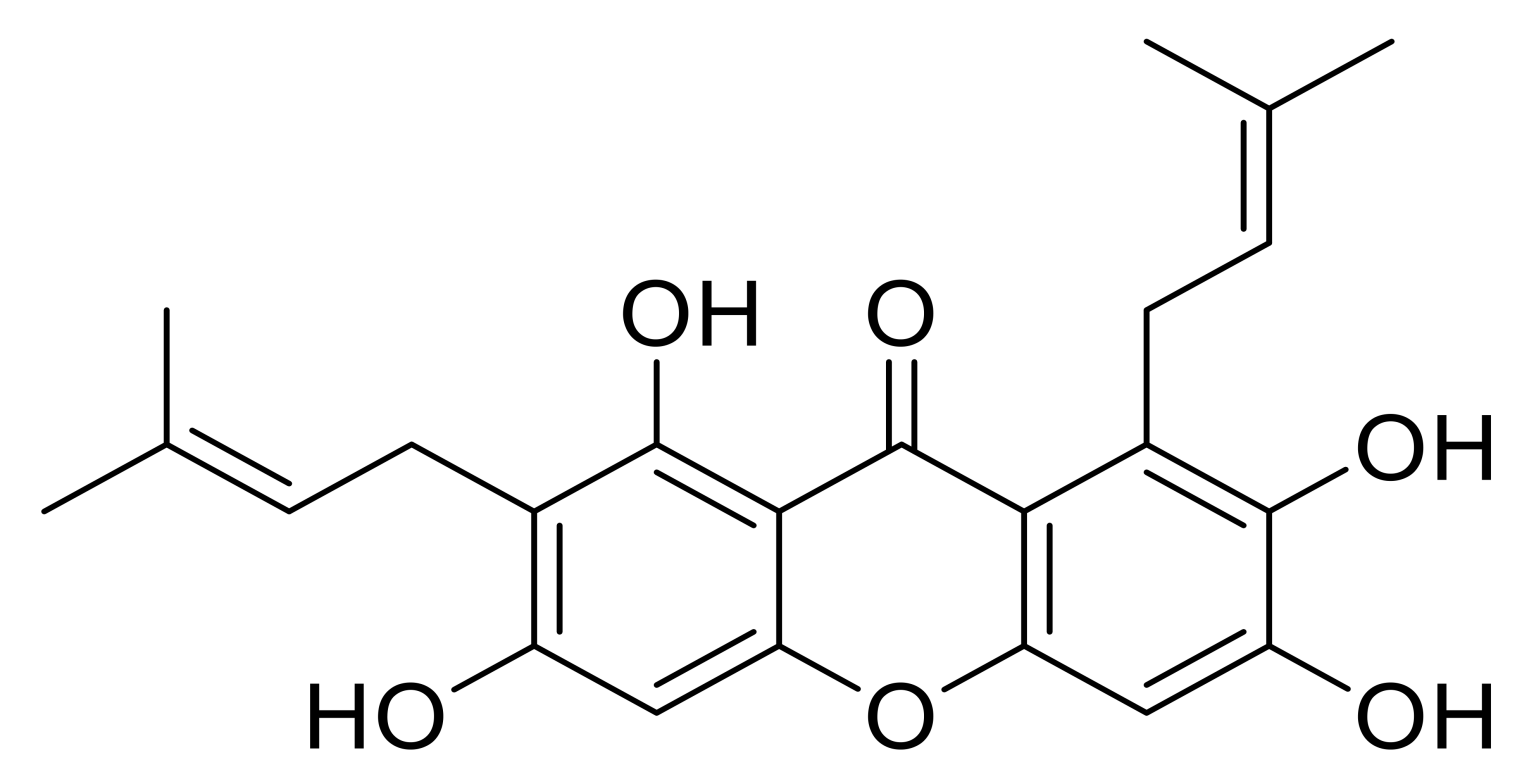	Inhibition of mRNA expression of MMP-1 and MMP-9, increase in TIMP-1 level; Modulation of the Nrf2-ARE signaling pathway; Upregulation of genes involved in skin structure and hydration, including COL1A1, ELN, and HAS-2; Downregulation of MAPKs, AP-1, and NF-κB; Autophagy induction by inhibiting mTOR and upregulating autophagy markers such as LC3-II, Beclin-1, and Atg5 [55]
Hyaluronic acid (HA) 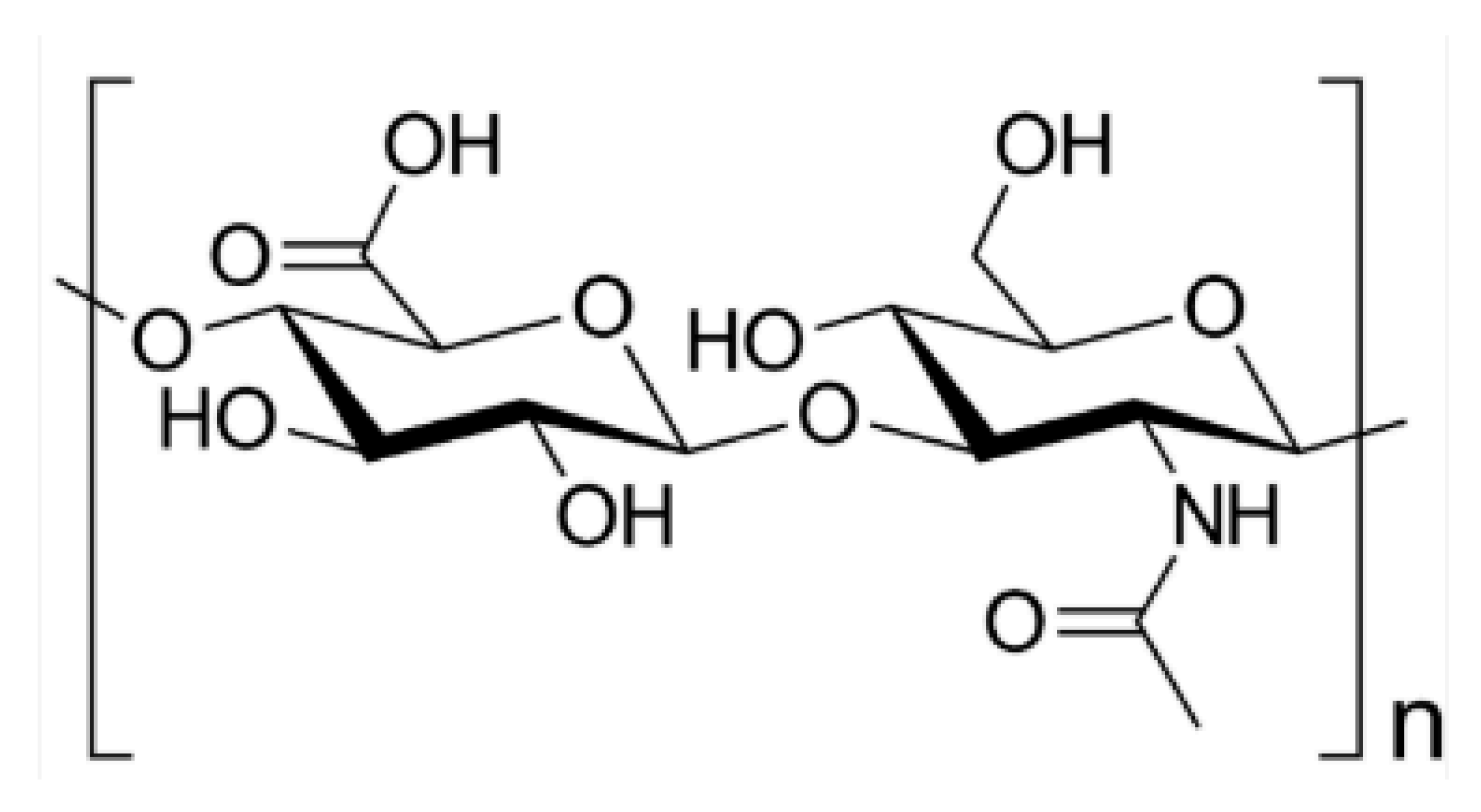	Upregulation of CD44 expression at different cellular levels, promoting the selective expression of the CD44v3 isoform in keratinocytes; Stimulation of keratinocyte proliferation in vivo in mouse models (showed by HaFi); Improved skin thickness and a subjectively reported soothing effect in human volunteers after topical administration (for HAFi); Filling, hydrating and viscoelastic properties showed by native non-fragmented HA (1000 kDa) [42,56,57,58]
Kinetin (N6-furfuryladenine) 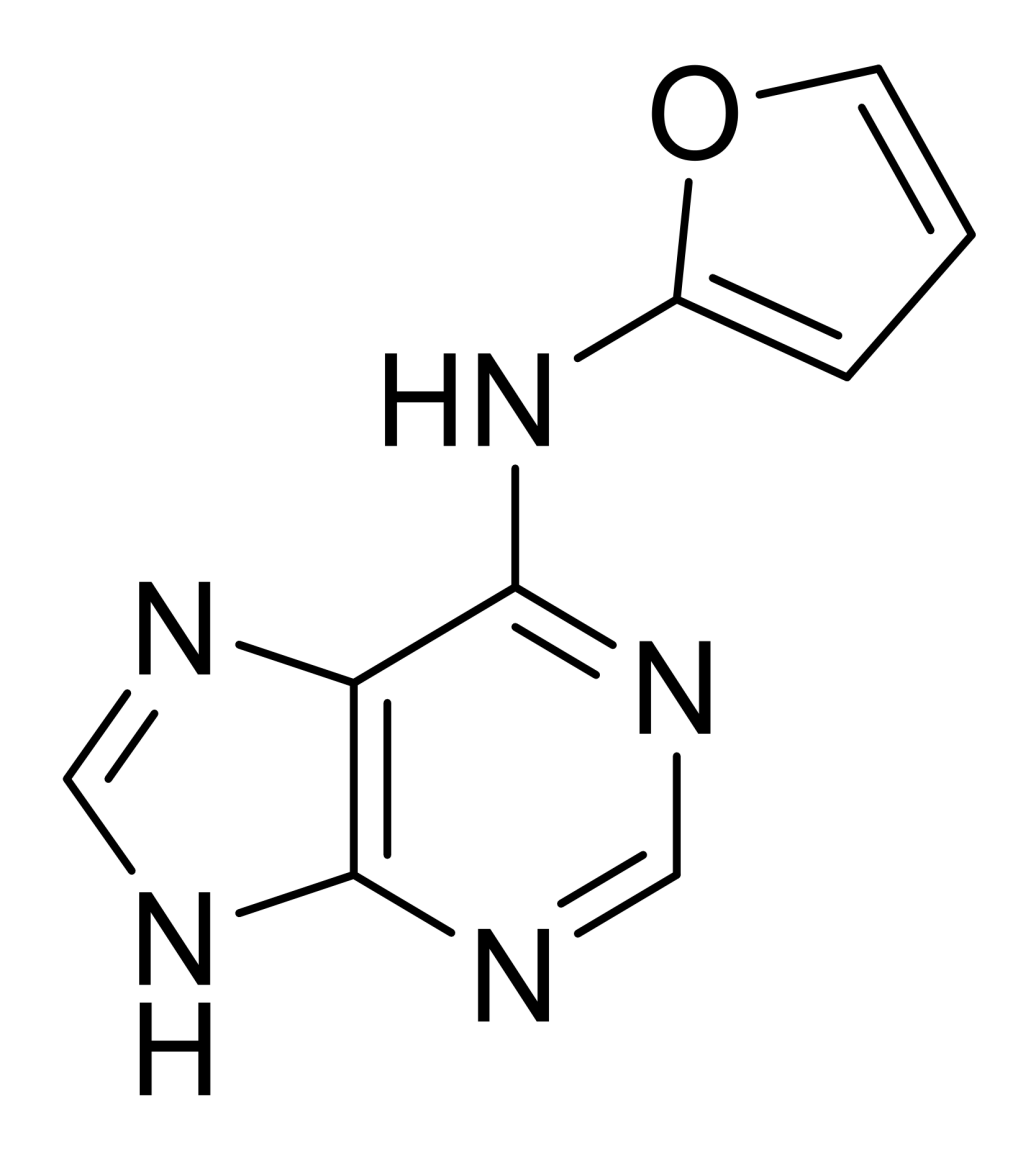	Xenobiotic ligand for CD44 adhesion receptor; Stimulation of the cell proliferation, increasing the activity of glutathione peroxidase and glutathione reductase in human dermal fibroblasts; Improvement of skin moisture levels with reduction in transepidermal water loss in vivo, effect on maintaining the skin’s barrier functions in atrophic skin [59,60]
Oleanolic acid 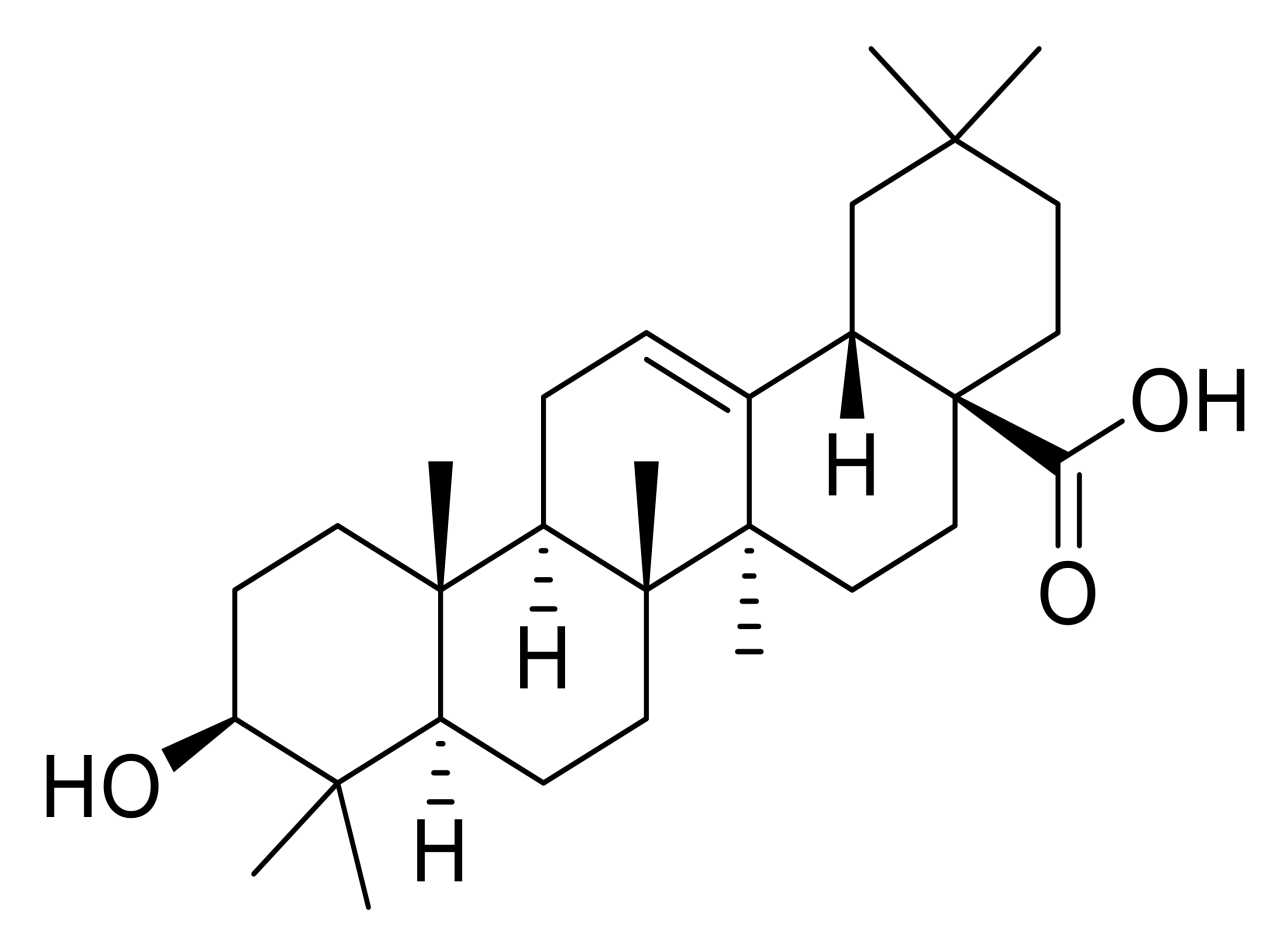	Activation of telomerase proved in human peripheral blood mononuclear cells; Antioxidant and anti-inflammatory activity; Cytoprotective effect on human keratinocytes [29,61,62]

AP-1—activator protein-1, ARE—antioxidant response element, COL1A1—collagen type I alpha 1 chain, ELN—elastin, HAFi—hyaluronate fragments of intermediate size, HAS-2—hyaluronan synthase 2, IL—interleukin, INCI—International Nomenclature of Cosmetic Ingredients, MAPKs—mitogen-activated protein kinases, NF-κB—nuclear factor kappa-light-chain-enhancer of activated B cells, Nrf2—nuclear factor erythroid 2-related factor 2, MMP—matrix metalloproteinase, ROS—reactive oxygen species, TIMP—tissue inhibitor of metalloproteinases.

### 2.3. Matrix Metalloproteinases

In recent years, growing attention has been paid to matrix metalloproteinases (MMPs) as molecular targets in anti-aging and skin-repairing cosmetic formulations. It was in the 1990s that MMPs were first identified as critical contributors to skin aging, marking a significant advancement in the understanding of the molecular mechanisms underlying age-related changes in the skin [63,64]. Matrix metalloproteinases (MMPs) are enzymes belonging to the family of metalloproteases that are dependent on metal ions (primarily zinc), playing a crucial role in the degradation of the extracellular matrix (ECM) and tissue remodeling. MMPs are produced by skin cells, such as fibroblasts, keratinocytes, macrophages, endothelial cells, mast cells, and eosinophils [65]. They are involved in numerous physiological and pathological processes, such as wound healing, embryonic development, angiogenesis, as well as the progression of diseases like cancer, inflammation, and cardiovascular disorders [66,67]. Considering that the components of the extracellular matrix provide essential structural and functional support to skin tissue, skin aging is closely associated with an imbalance between their synthesis and degradation. MMPs, which are primarily responsible for the degradation of key structural proteins such as collagen and elastin, play a pivotal role in this process [68]. The expression of matrix metalloproteinases is upregulated by various factors such as UV radiation, inflammatory cytokines, oxidative stress, and certain growth factors [63,64,69].

When considering matrix metalloproteinases as molecular targets in modern cosmetic formulations, it is essential to also mention their natural regulators—the tissue inhibitors of metalloproteinases (TIMPs). TIMPs are specialized proteins that regulate the activity of MMPs in tissues, helping to control their localized functions [70]. TIMPs regulate the proteolytic activity of MMPs by direct interaction with these enzymes, and not by regulation at a transcriptional level. A disruption in the balance between MMP and TIMP activity is necessary to initiate excessive extracellular matrix breakdown, which in turn promotes tissue remodeling. It was shown that TIMP-1, which inhibits most MMPs, plays a crucial role in protecting human skin from photodamage and photoaging, and the expression of TIMP-1 is significantly decreased both in photoaged and intrinsically aged skin [71,72].

To date, a number of compounds with anti-aging properties have been identified, targeting MMPs as their primary point of action. These include both direct MMP inhibitors and substances that modulate signaling pathways involved in the upregulation of MMP expression—collectively referred to in the literature as classical metalloproteinase inhibitors. Following the discovery of the link between MMPs and skin aging, Fisher demonstrated that retinoic acid exerts anti-aging effects by inhibiting MMP expression and promoting the synthesis of extracellular matrix components such as collagen [63]. Since then, researchers have been searching for more effective compounds that are more selective, less irritating, and more stable in cosmetic formulations. Matrix metalloproteinase inhibitors have been recently discussed as potential components of anti-aging cosmetic products in a comprehensive review by Li et al. [69].

Among anti-aging ingredients, the tripeptide GHK-Cu (glycyl-L-histidyl-L-lysine-copper; INCI: Copper Tripeptide-1) stands out for its dual regulatory effect on MMPs and TIMP. It has been shown that GHK-Cu increases mRNA expression of MMP-1, MMP-2, and TIMP-1. The biological effect of GHK-Cu include stimulation of collagen and elastin production, and reduction in wrinkle volume (Table 1) [48]. Another noteworthy ingredient is Tripeptide Palmitoyl RGD (palmitoyl-Arg-Gly-Asp). Found in various extracellular matrix components, it is crucial for cell adhesion. In vitro studies showed that it activates type I procollagen production and suppresses MMP-1. A 12-week clinical study on Palm-RGD cream demonstrated significant wrinkle reduction and improved skin roughness. Further analysis revealed Palm-RGD positively impacts skin elasticity and notably increases dermal density [73].

While traditional retinoids and peptides remain important, one of the most innovative anti-aging approaches targeting MMPs focus on the use of a novel form of a peptide nucleic acid (PNA) derivative—PNA-20 Carboxyethyl fluorene (PNA-20 CEF). PNA-20 CEF comprises 14 N-(2-aminoethyl)glycine monomers with a specific sequence (Fethoc-TACTCACCATATAT-NH2), featuring six modified nucleobases that enable predictable and complementary hybridization with target sequences. The molecule acts on human MMP-1 pre-mRNA at its exon-intron junction. By binding to this specific site, PNA-20 CEF effectively induces exon skipping during the pre-mRNA splicing process. This manipulation results in the production of a non-functional MMP-1 protein [74].

In vitro studies with human dermal fibroblasts showed that PNA-20 CEF significantly reduces both MMP-1 gene expression and protein concentration in a dose-dependent manner. PNA-20 CEF treatment significantly increased collagen I protein expression in human dermal fibroblasts. Furthermore, PNA-20 CEF was shown to penetrate reconstructed human 3D skin tissue from the epidermis into the upper dermis. In vitro studies with human dermal fibroblasts also demonstrated that a cosmeceutical cream containing PNA-20 CEF at 0.05% significantly reduced MMP-1 gene expression and protein levels while concurrently increasing collagen I propeptide (PIP1 α1) expression, mirroring the effects observed with the isolated compound. Clinical study involving daily application of cream containing PNA-20 CEF proved its beneficial effect on skin density, moisture, and elasticity, as well as wrinkle reduction [74].

The epigenetic modulation of enzyme carnitine acetyltransferase (CRAT) seems to be an interesting molecular target associated with MMPs. It was shown that CRAT is downregulated by ultraviolet exposition via promoter hypermethylation. It contributes to the activation of ERK, JNK, and p38 MAPK signaling pathways, and then increasing of MMP-1 expression. This suggest that controlling CRAT through epigenetic changes could be a new way to treat photoaging [75].

### 2.4. Antioxidant Enzymes

Oxidative stress contributes to skin aging by affecting multiple signaling pathways, leading, among others, to the activation of matrix metalloproteinases, which induce and accelerate the degradation of skin structure [76]. Reactive oxygen species (ROS) stimulate MAPKs and play a role in the regulation of transcription factors such as NF-κB and AP-1, which, as mentioned above, lead to the increased expression of MMPs and downregulate the expression of TIMPs. In the context of anti-aging effects, the use of cosmetic ingredients with strong antioxidant capacity is particularly important.

It was shown that the modulation of the Nrf2-ARE pathway is recognized as one of the central mechanisms underlying the anti-aging effects of various compounds. The Nrf2-ARE signaling pathway is activated by ROS, and as a result, the expression of antioxidant enzymes is upregulated; thus, it plays a crucial role in protecting skin cells from oxidative damage [77]. Under normal conditions, Nrf2 (nuclear factor erythroid 2-related factor 2) is kept inactive in the cytoplasm by its interaction with inhibitor protein Keap1 (Kelch-like ECH-associated protein 1), which plays a key role in regulating Nrf2 activity. In stressful conditions reactive oxygen species modify Keap1, allowing Nrf2 to detach and translocate into the nucleus. Once in the nucleus, Nrf2 binds to antioxidant response elements (ARE), promoting the expression of protective antioxidant enzymes like heme oxygenase-1 (HO-1) [78]. γ-Mangostin (Table 1), a xanthone derivative, serves as an example of an anti-aging compound of which its mechanism of action involves, among others, the modulation of the Nrf2-ARE signaling pathway [55].

### 2.5. mTOR Pathway

The mechanistic target of rapamycin (or mammalian target of rapamycin, mTOR) is a serine/threonine protein kinase that functions as a master regulator of cellular growth and metabolism [79]. The inhibition of the mTOR pathway has been experimentally shown to extend lifespan in various animal models and protect against a growing range of age-related diseases. Rapamycin (sirolimus), an FDA-approved immunosuppressant, functions as an mTOR inhibitor. Numerous studies have confirmed its ability to extend lifespan in multiple species, including yeast, nematodes, fruit flies, and mice [80,81]. In a clinical study involving 40 participants, it was shown that topical application of 10 μM rapamycin for 8 months significantly influenced both the molecular markers of cellular senescence and visible signs of skin aging [82]. Metformin is used for treatment type 2 diabetes. Beyond its glucose-lowering effects, metformin plays a promising role in aging research by modulating key longevity pathways: mTOR, AMPK, and SIRT1 [83]. In vitro studies have confirmed that metformin can attenuate UVA-induced photoaging, in part by modulating the PI3K/AKT/mTOR signaling pathway [84].

One of the crucial processes regulated by mTOR is the initiation of autophagy. It acts via the inhibition of autophagosome formation and autophagy genes. Autophagy eliminates damaged organelles and macromolecules from the cytoplasm, while also allowing cells to reuse amino acids, particularly during nutrient scarcity [85]. Under conditions of oxidative stress, autophagy contributes to cellular protection by degrading down oxidized proteins and lipids, thereby reducing oxidative damage [76]. A lack of efficient autophagy causes cellular damage to accumulate, ultimately contributing to senescence [86]. Rahmouni et al. linked the mTOR pathway to sagging skin [87]. Disruptions in mTOR signaling pathway have been linked to various skin-related disorders, such as delayed wound healing, atopic dermatitis, and the other hyperproliferative skin disorders [88].

### 2.6. AMPK Pathway

Modulation of AMP-activated dependent kinase (AMPK) positively influences cellular resistance to stress, inflammation, and aging. AMPK, alongside mTOR and SIRT1, forms a network that links cellular metabolism and longevity programs [81,83]. AMPK is activated in response to an increase in AMP/ATP ratio and inhibits mTORC1. It was shown that overexpression of AMPK extends the lifespan in *Caenorhabditis elegans*. Additionally, metformin that activates AMPK has been shown to prolong lifespan in both *C. elegans* and in short-lived, cancer-prone strains of mice [79,89]. In the studies with nasal polyp–derived fibroblasts, activation of AMPK by metformin contributed to the decrease in the expression of MMP-1 and MMP-2 [90]. The ability to activate AMPK is associated with the anti-aging effects of extracellular superoxide dismutase [91]. Moreover, the mechanism of anti-aging action of resveratrol is associated with its ability to activate of AMPK, which in turn promotes autophagy, reduces oxidative stress, inhibits apoptosis, and restores normal cell cycling [92].

### 2.7. CD44 Adhesion Receptor

Age-related skin thinning and loss of dermal integrity are important features of intrinsic skin aging and are associated with changes in the extracellular matrix, including alterations in hyaluronic acid (HA) metabolism and connective tissue organization. These changes contribute to reduced skin hydration, elasticity, and firmness and consequently to the development of visible signs of skin aging. Molecular pathways involved in the regulation of HA metabolism and its interaction with cell-surface receptors such as CD44 may therefore represent relevant targets for cosmetic strategies aimed at maintaining skin hydration and supporting skin structure during aging [56,93,94,95].

CD44 is a transmembrane glycoprotein belonging to the family of cell adhesion receptors and is expressed in various skin cell types, including keratinocytes and fibroblasts. The receptor interacts with components of the extracellular matrix and serves as a major cell-surface receptor for hyaluronic acid, contributing to its retention at the cell surface and mediating the internalization of HA and other glycosaminoglycans. Due to their hydrating and skin-conditioning properties, HA and related glycosaminoglycans are widely incorporated into cosmetic formulations, particularly products intended to improve skin hydration, elasticity, and the appearance of visible signs of skin aging.

The interaction between HA and CD44 contributes to the regulation of several cellular processes relevant to skin homeostasis, including keratinocyte proliferation, differentiation, and migration. Both HA metabolism and CD44 expression may be affected by environmental factors, including ultraviolet radiation, which can contribute to alterations in skin hydration and structural integrity during skin aging [56,96,97,98]. HA metabolism is regulated by hyaluronan synthases (HAS1, HAS2, and HAS3) and hyaluronidases, which determine the amount and molecular size of HA present in the extracellular environment. The biological effects of HA are strongly dependent on its molecular weight, and this aspect is particularly relevant when selecting HA for cosmetic formulations. CD44 exists in several isoforms generated by alternative splicing and post-translational modifications, with CD44v3 being expressed in keratinocytes and associated with HA-dependent regulation of epidermal cell behavior [99,100].

The potential cosmetic relevance of CD44–HA interactions has been investigated using HA fragments of defined molecular weight. Intermediate-molecular-weight HA fragments (HAFi; 50–400 kDa) have been reported to influence keratinocyte proliferation and epidermal thickness in experimental models. In a study involving human volunteers, topical application of 1% HAFi was associated with improvements in skin thickness and a subjective soothing effect, whereas comparable effects were not observed following application of low- or high-molecular-weight HA [56]. These findings suggest that the molecular size of HA may influence its interaction with CD44 and consequently its biological effects in the skin, providing a rationale for the development of cosmetic formulations containing HA of defined molecular characteristics.

From a cosmetic perspective, the CD44–HA axis represents a promising molecular target for the development of products aimed at maintaining skin hydration, supporting epidermal homeostasis, and improving the appearance of aging skin. However, the broad distribution and multifunctional nature of CD44 highlight the importance of evaluating the biological effects and safety of ingredients designed to influence this pathway. In particular, the molecular weight and structural characteristics of HA should be considered when developing HA-based cosmetic formulations, as different HA fractions may interact differently with CD44 and other HA-binding receptors [101,102,103].

Naturally derived compounds such as resveratrol and kinetin (Table 1) have also been investigated for their potential cosmetic applications in skin care. Resveratrol is a stilbene-derived polyphenol naturally occurring in grapes, berries, tea, and peanuts. Its biosynthesis in plants is induced by various stress conditions, including tissue injury, infection, and UV exposure [92,104]. Kinetin is a cytokinin and adenine derivative naturally occurring in plant cells. Both compounds have attracted interest in cosmetic research because of their potential antioxidant and skin-conditioning properties. Kinetin has also been investigated for its ability to influence cellular processes relevant to skin appearance and homeostasis [105].

## 3. Molecular Targets in the Search for Novel Melanogenesis Inhibitors

**Figure 1:**
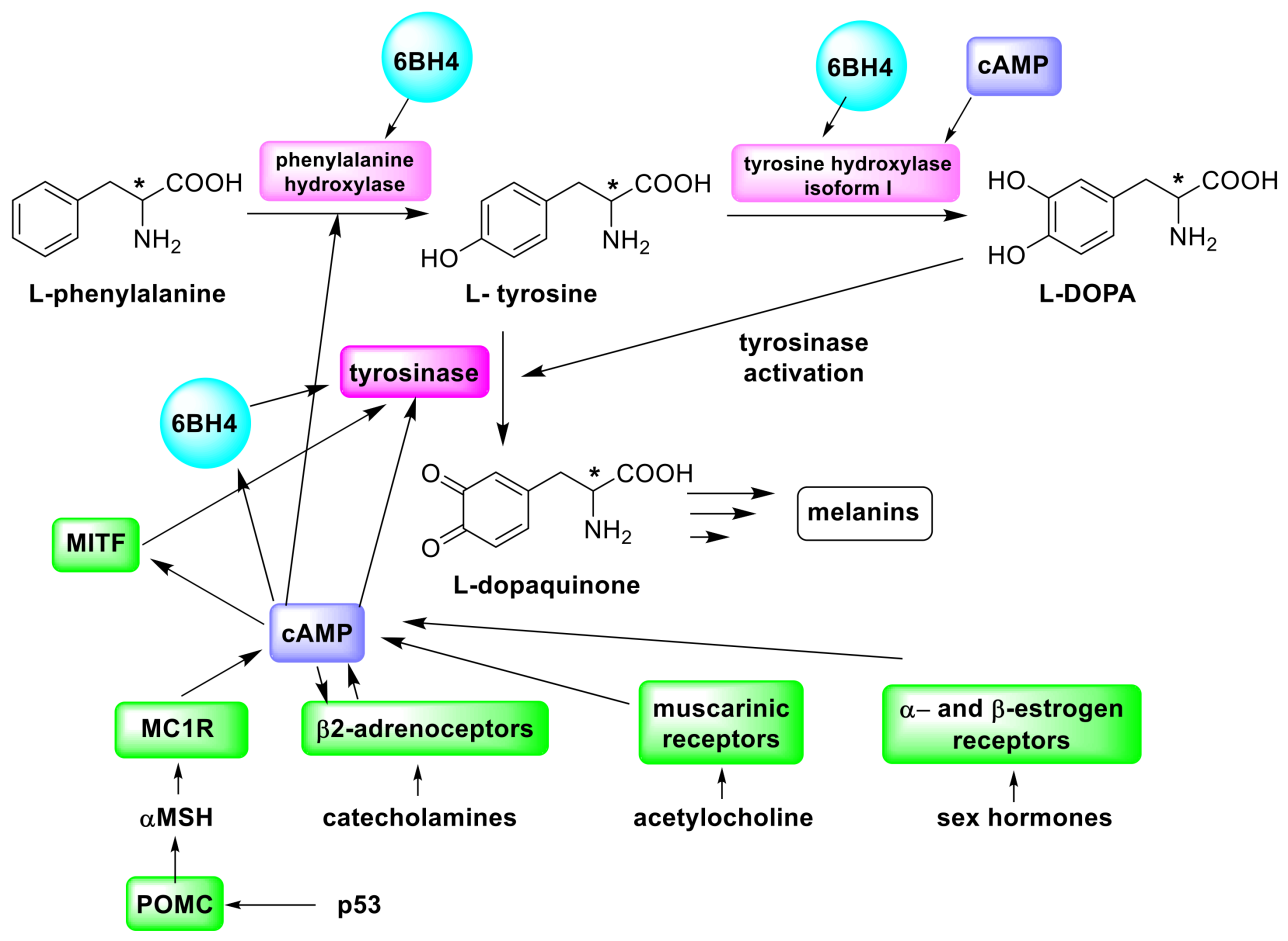
First steps of melanogenesis process with potential molecular targets of cosmetic ingredients used in hyperpigmentation disorders (α-MSH—alpha-melanocyte stimulating hormone, cAMP—cyclic-3′,5′-adenosine monophosphate, MITF—microphthalmia-associated transcription factor, MC1R—melanocortin 1 receptor, 6BH4—6(R)-L-erythro-5,6,7,8-tetrahydrobiopterin, POMC—proopiomelanocortin), *—asymmetric carbon atom.

Melanogenesis inhibitors are used in bleaching (depigmenting) cosmetic formulation in order to support the treatment of hyperpigmentation disorders such as melasma, lentigines, and café-au-lait, post-inflammatory, solar, drug-induced, and hormonal hyperpigmentations. Hyperpigmentation constitutes an important esthetic concern significantly affecting patients’ quality of life [106,107,108]. Tyrosinase represents the classical and most extensively investigated molecular target in the development of melanogenesis inhibitors, as it catalyzes key enzymatic steps in melanin biosynthesis (Figure 1). Correlations between the inhibition of tyrosinase and pigmentation have been observed, which proved the effectiveness of the strategy [109]. However, melanogenesis is regulated by a complex network of upstream signaling pathways and transcriptional regulators that control tyrosinase expression and activity. Among these, the MC1R/cAMP/PKA/CREB/MITF axis plays a central role in the regulation of melanogenic gene expression, while additional targets, including β2-adrenoreceptors, sex hormone receptors, and 6(R)-L-erythro-5,6,7,8-tetrahydrobiopterin (6BH4), have also emerged as potential targets for cosmetic modulation of pigmentation [110]. Melanogenesis is also regulated by other internal and external factors including p53, tyrosine hydroxylase isoform I, proopiomelanocortin (POMC)-derived peptides, and also UV radiation (Figure 1), and despite intensive work, these mechanisms are still not fully understood, giving rise to the search for more efficient depigmenting agents in the future [111].

### 3.1. Classical Target: Human Tyrosinase (hsTYR)

Human tyrosinase belongs to the tyrosinase family (TYR, EC 1.14.18.1) together with enzymes from plants, fungi, bacteria, and other mammals. It is a metalloenzyme with two copper atoms in its active site, coordinated with histidine residues. This region is highly conserved among tyrosinases, but enzymes from various sources are actually very different. Human tyrosinase significantly differs from *Agaricus bisporus* tyrosinase (main differences are presented in Table 2); in fact, they only share 23% sequence identity, so using mushroom tyrosinase in the search for depigmenting agents in humans is considered not effective anymore [109,112].

The shortcomings in the use of human tyrosinase in the research resulted primarily from the lack of an appropriate expression system in which enzymes could be produced and then purified. The problem resulted also from difficulties in obtaining adequately large amounts of protein. As expression systems, only cells able to produce proteins with post-translational modifications, including glycosylation, can be used, excluding *Escherichia coli*. Extensive works have led to expression of the full-length human TYR in *Spodoptera frugiperda* ovary cells (Sf9 line). The obtained enzyme was successfully used in the reactions with substrates and inhibitor molecules [116]. Because *hs*TYR is a transmembrane protein, the strategy for possible simplifying the handling of protein is deleting the membrane-anchoring helix. This strategy was implemented to obtain the intra-melanosomal domain of *hs*TYR in *Trichoplusia ni* insect larvae infected with the baculovirus vector. Then the protein was successfully purified from whole-insect larval biomass. The activity of this recombinant enzyme was confirmed to be the same as tyrosinase in vivo function [117]. The expression and extraction of recombinant proteins from insect larvae is time-consuming and requires multiple purification steps, which lowers the yield of the production. Large-scale recombinant expression of *hs*TYR was, however, reported in High Five cells derived from *Trichoplusiani* using the baculovirus expression vector system. An N-terminal honeybee melittin signal peptide was fused with the encoding sequence, which resulted in the enhancement of secretion of the produced protein into the medium. In this study only two purification steps were employed to obtain highly pure protein samples at a sufficient yield (4–6 mg per liter of culture) for crystallization trials and screening of skin whitening agents [113].

**Figure 2:**
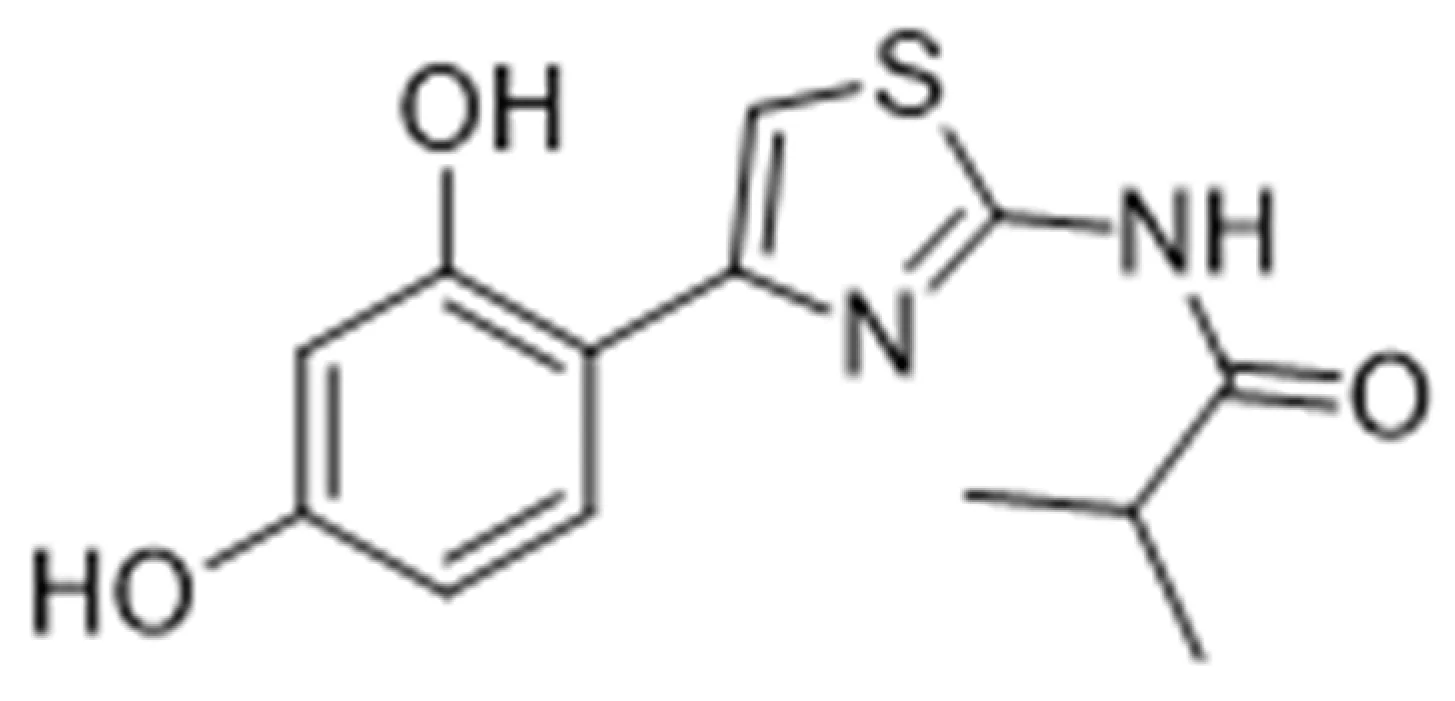
Chemical structure of thiamidol (INCI: Isobutylamido thiazolyl resorcinol) melanogenesis inhibitor identified by HTS using *hs*Tyr produced in HEK 293 cells.

Even well-established melanogenesis inhibitors used in cosmetic products such as kojic acid, hydroquinone and arbutin showed no significant effect against the human enzyme. On the contrary, one of the most recently identified melanogenesis inhibitors, thiamidol (INCI: Isobutylamido Thiazolyl Resorcinol, Figure 2), was selected in High-Throughput Screening (HTS) using human tyrosinase produced in genetically modified HEK 293 cells. It did not effectively inhibit the mushroom enzyme and could fail in the development process in which mushroom tyrosinase is used [118,119]. In this experiment, a His-tagged soluble form of *hs*Tyr expressed in HEK 293 cells and purified by metal affinity chromatography was used. This active protein comprised the catalytic domain of *hs*Tyr and had the same catalytic properties as wild-type *hs*Tyr [120]. Thiamidol, apart from inhibition of *hs*Tyr in vitro, showed significant clinical efficacy [118,119].

Life cell-based tyrosinase assays in mouse melanoma or human melanoma cells are also used to evaluate the effectiveness of whitening agents. These assays require long incubation of the potential inhibitor with cells (from 48 h up to 5 days). As a useful alternative, an assay using tyrosinase obtained from human melanoma MM418C1 cell lysate was proposed. This assay enables the screening of novel potential skin whitening agents within 3 h after collecting cell pellets [121]. Recently, human tyrosinase displayed on the surface of Chinese hamster ovary cells for ligand fishing of tyrosinase inhibitors from medicinal plants was also reported [122].

### 3.2. Upstream Regulation of Melanogenesis: MC1R/cAMP/PKA/CREB/MITF

Cyclic-3′,5′-adenosine monophosphate (cAMP) was shown to play a central role in the control of tyrosinase expression, and it also induces expression of tyrosine hydroxylase, as well as regulates phosphorylation of the SER16 in phenylalanine hydroxylase in protein kinase A pathway [123,124]. cAMP is synthesized by adenylylcyclase coupled to several G-protein receptors, including the well-known melanocortin 1 receptor (MC1R) for alpha-melanocyte stimulating hormone (α-MSH), but also the β2-adrenoceptor for catecholamines, the muscarinic receptors for acetylcholine, and the α-and β-estrogen receptors for sex hormones. Moreover, corticotropin-releasing factor (CRF) via the CRF-R1 signal promotes cAMP synthesis and stimulates, in that pathway, proopiomelanocortin (POMC) and adrenocorticotropin (ACTH) production [125,126]. Activation of MC1R and other G protein-coupled receptors increases intracellular cAMP levels, which activates protein kinase A (PKA) and subsequently promotes CREB phosphorylation. This signaling cascade enhances MITF expression and activity, thereby increasing the transcription of melanogenic genes, including TYR. Thus, MC1R, cAMP, PKA/CREB, and MITF represent interconnected upstream regulatory targets that ultimately influence tyrosinase expression and melanogenesis.

Microphthalmia-associated transcription factor (MITF) seems to be the most important transcription factor in the regulation of tyrosinase expression. Mutations of MITF gene in human cause skin and hair depigmentation. After UV exposure a well-characterized MSH/MC1R-cAMP-MITF pathway is activated, significantly inducing melanin production. However, MITF was proved to be required but not sufficient to induce the expression of melanogenic genes [127,128]. Inhibition of MITF expression is already an accepted way for the identification of novel melanogenesis inhibitors. Several bioactive compounds have already been found to inhibit MITF expression, including derivatives of cinnamic acid [129,130], paenol [131], and coumarin [132]. Some of them were proposed as novel cosmetic ingredients for hyperpigmentation. However, more precise evaluation of molecular targets is usually required. For example, diacetylcaffeic acid cyclohexyl ester was found to decrease MITF expression by direct impact on the nuclear import of cAMP-regulated transcriptional co-activator 1 (CRTC1) in melanocytes [133]. An oligostilbene component of *Caragana sinica*—α-viniferin—inhibits cAMP/PKA-signaled phosphorylation of CREB, coupled with dephosphorylation of CRTC1, resulting in the downregulation of expression of MITF and tyrosinase genes. The melanogenesis inhibition was proved for this compound in vitro in cAMP-elevated melanocyte cultures as well as in vivo in a double-blind, vehicle-controlled, split-face human trial [134]. Decursin was proved to prevent melanogenesis by suppressing MITF expression through the regulation of PKA/CREB, MAPKs, and PI3K/Akt/GSK-3β cascades. The in vitro anti-melanogenic activity for this compound was proved in reconstructed human skin tissue [132].

### 3.3. Control of Skin Pigmentation by 6(R)-L-Erythro-5,6,7,8-Tetrahydrobiopterin

6(*R*)-L-erythro-5,6,7,8-tetrahydrobiopterin (6BH4) contributes to melanogenesis process in few aspects. Appropriate cAMP response elements (CREs) localized in promoter regions of melanogenesis-related genes control the initial step for 6BH4 synthesis via guanosine-5′-triphosphate (GTP) cyclohydrolase [135]. Under physiologic conditions, undifferentiated human epidermal keratinocytes and melanocytes are able to de novo-synthesize, recycle, and regulate 6BH4. 6BH4 is an L-phenylalanine hydroxylase (PAH) cofactor and electron donor, enabling the conversion of L-phenylalanine to L-tyrosine, as well as enabling the conversion of L-tyrosine to L-DOPA. In that aspect, 6BH4 seems to control the whole melanogenesis process [111,136]. Moreover, 6BH4 was shown to uncompetitively inhibit tyrosinase [137]. Epidermal 6BH4 levels as well as PAH activities correlate linearly with skin phototypes; in dark skin its activity was shown to be the highest. Taking into consideration the above facts, 6BH4 is regarded as a major player in the regulation of skin color and may become a new molecular target for cosmetic depigmenting ingredients [111].

### 3.4. β2-Adrenoceptors and Estrogen Receptors

It was shown that β2-adrenoreceptors present in melanocytes membranes significantly contribute to increasing of the melanogenesis process. Activation of these receptors causes synthesis of cAMP; additionally, these receptors possess CRE binding domains in their promoter regions, and also 6BH4 regulatory binding sites, which in turn increase receptor densities and cAMP levels [138]. Based on these facts, one of the potential strategies for the treatment of hyperpigmentation is the inhibition of β2-adrenoreceptors. Similarly, signals transduced by estrogen receptors notably increase melanogenesis [139,140], and inhibition of these receptors could also possibly contribute to hyperpigmentation treatment.

## 4. Molecular Targets for Sunless Tanning

Contrary to the ongoing search for novel melanogenesis inhibitors, a considerable number of consumers actively seek and prefer cosmetic ingredients that enhance skin pigmentation or promote skin darkening. These cosmetic ingredients represent also a potential strategy for skin cancer prevention [128] as well as potential treatment options for vitiligo. The classical sunless tanning agent used in the beauty industry is a monosaccharide, ketose, and triose—dihydroxyacetone (1,3-dihydroxypropan-2-one). This compound is able to form brown melanoidins as a result of a non-enzymatic Maillard reaction initiated by a condensation of its carbonyl group with an amine group of amino acid residues found in the *stratum corneum*. Despite several limitations of its use, such as undesirable color tones, formulations challenges and safety concerns, it still constitutes the main tanning agent in sunless tanners [141,142]. In addition, several naturally derived compounds have been investigated as tanning agents, including juglone (5-hydroxy-1,4-naphthoquinone), derived from walnut (*Juglans regia*) tissues, which can produce a brownish coloration through reactions with skin proteins and has therefore attracted interest as a potential natural alternative to conventional sunless tanning agents. Other plant-derived compounds, such as lawsone from henna (*Lawsonia inermis*), may similarly provide a temporary skin-darkening effect through interaction with keratin [143]. Further research is conducted to provide novel alternatives targeting various intramolecular structures, with particular emphasis on safety and epidermis penetration issues.

### 4.1. cAMP Induction

There are known compounds which act in melanocytes as melanogenesis inducers *via* the cAMP pathway. As shown in Figure 1, cAMP plays important role in the melanogenesis contributing to several steps of the process. Forskolin, a diterpene found in the roots of *Plectranthus barbatus*, was shown to increase cytoplasmic cAMP levels by direct activation of adenylyl cyclase. Topical application of this compound was postulated to effectively promote production of eumelanin [144]. Another concept arose from inhibition of the cAMP-degrading enzyme—phosphodiesterase (PDE), and more specifically PDE4D3, which was identified as a key regulator of cAMP homeostasis in melanocytes. Tests in mice showed that topical application of a PDE4D3 inhibitor resulted in UV-independent hyperpigmentation similar to the effect of forskolin treatment [145].

### 4.2. Melanocortin Receptor

As alpha-melanocyte stimulating hormone (α-MSH) is a major internal factor inducing melanogenesis primarily *via* targeting melanocortin receptors, Rational design strategy for novel tanning agent focused on synthetic α-MSH analogs. A significant example of a molecule with this mechanism of action is afamelanotide (Melanotan I). It is a thirteen amino acid linear peptide (Ac-Ser-Tyr-Ser-Nle-Glu-His-D-Phe-Arg-Trp-Gly-Lys-Pro-Val-NH_2_) with specific modifications, including the replacement of methionine by norleucine (Nle), and L-phenylalanine by D-phenylalanine. These changes make it more resistant to enzymatic degradation than its natural analog.

Afamelanotide mimics the action of the natural hormone on melanocortin receptors, leading to a variety of effects. It increases melanin production by activation of the MC1R receptors and represents an example of a strategy for achieving skin darkening through direct interaction with specific molecular targets involved in melanogenesis. It can also affect other related physiological functions by possible interactions with MC2R, MC3R, MC4R, and MC5R receptors. However, the structural modifications in the molecule resulted in increased specificity for MC1R and made Melanotan I a more targeted agent for stimulating melanin production with fewer side effects [146].

In addition to their roles in skin and hair coloring, melanocortin 1 receptors have gained attention for their anti-inflammatory activities. The melanocortin 1 receptor serves as a promising therapeutic target for a range of conditions, including melanoma, vitiligo, and systemic sclerosis. Following UV-induced skin damage, melanocortin peptides such as α-MSH and ACTH are produced in the skin and exert protective effects by binding to MC1R and initiating downstream signaling changes. MC1R also demonstrates notable anti-inflammatory properties, and variants in the MC1R gene are known to contribute significantly to differences in human skin color. More recently, MT-7117 has emerged as a highly selective MC1R agonist, demonstrating substantially greater receptor specificity compared to the reference compound NDP-MSH. Another compound of interest, camptothecin-MT-II, showed an IC50 of 16 nM, suggesting its potential utility in overcoming drug resistance in cancer treatment. These findings underscore the therapeutic versatility of MC1R-targeted strategies and highlight the receptor’s central role in both pigmentation biology and broader disease management [147,148].

**Figure 3:**
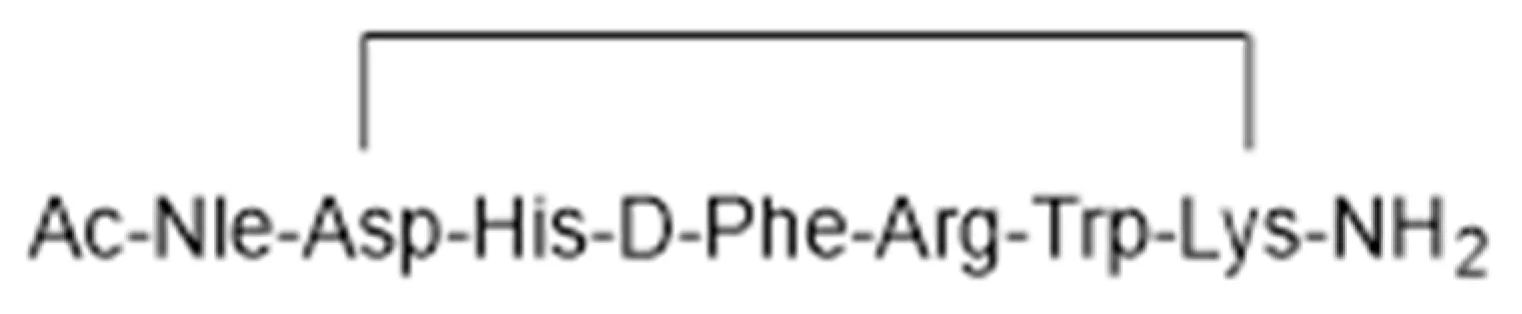
Amino acid chain of Melanotan II.

Afamelanotide is approved in Europe and the USA for the treatment of erythropoietic protoporphyria (EPP) in adult patients. It has been shown to improve tolerance to artificial white light and to extend the duration of pain-free exposure to direct sunlight in patients with EPP. Potential dermatological applications of Melanotan I also include polymorphic light eruptions, solar urticaria, vitiligo (in combination with phototherapy), as well as rare and case-based conditions such as acne vulgaris, Hailey–Hailey disease, and reduction in DNA photodamage in the skin of patients with pigmentary disorders (xeroderma pigmentosum) [149]. Another synthetic α-MSH analog, Melanotan II, is a cyclic seven amino acid peptide with a lactam ring formed by an ε-amino group of lysine and an γ-carboxy group of aspartic acid (Figure 3) [146].

Melanotan II is more potent than natural α-MSH but, despite its high potency, exhibits lower receptor specificity than Melanotan I. It is characterized by broad agonistic activity on multiple melanocortin receptors (MC1R, MC3R, MC4R), which underlies its effects beyond pigmentation, also influencing appetite and libido. Originally, Melanotan II was studied for its photoprotective properties, but due to observations of systemic effects such as improved erectile function (it was investigated as a treatment for sexual dysfunction), it was ultimately not commercialized because of adverse effects, including hypertension. Melanotan II is banned in most countries due to its side effects but dominates the illegal market. On the black market, Melanotan is sold under the name “Barbie drug” due to its tanning, anorexigenic, and libido-enhancing effects. Unregulated use of substances that manipulate key molecular pathways carries serious risks. Non-specific activation of multiple melanocortin receptors by Melanotan II can lead to a cascade of adverse molecular and physiological effects, including hypertension, the appearance of pigmented lesions (nevi), increased melanocyte proliferation, and reduced inflammatory and immune responses. Other reported side effects include nausea, vomiting, anxiety, facial flushing, chest pain, and kidney failure. Cases of melanoma have also been observed (although a causal relationship has not been definitively established), as well as changes in the appearance of existing moles. Since the drug is administered by injection, there is a risk of infection due to improper administration or sharing of needles. Black market products do not guarantee quality, safety, or efficacy. They may be mislabeled, contaminated, contain incorrect concentrations, or lack active ingredients [141,146,150,151,152]. For example, powder samples of Melanotan II seized have been found to be only 30% pure. Additionally, identification of Melanotan II is challenging for toxicologists because it is a large-molecular-weight peptide (1024 Da) and often carries multiple charges, which is unusual for typical drugs and narcotics. Mass spectrometer parameters may be limited to detecting masses up to 1000 Da. The UV spectrum of Melanotan II was initially unavailable in the literature, complicating its identification. Difficulties in detecting this substance may partly explain its popularity on the illegal market [153].

### 4.3. Salt-Inducible Kinase (SIK) Inhibition

SIK is known to regulate the activity of CREB-regulated transcription coactivator (CRTC), which, in turn, enhances MITF transcription. The concept of developing SIK inhibitors to promote melanogenesis has led to the discovery of small molecules that effectively stimulate melanin synthesis through this mechanism. Reported examples include YKL 06-061 and YKL 06-062, which were rationally designed to easily penetrate into melanocytes. Passive topical application of YKL 06-061 and YKL 06-062 to human skin explants induced significant pigmentation after 8 days of treatment [154].

## 5. Molecular Targets in Skin Disorders and Adnexal Care

### 5.1. Molecular Targets in Possible Intervention in Scars

Scars represent the final outcome of wound healing, a complex process consisting of successive phases. The first is the inflammatory phase, during which the immune system is activated, inflammatory cells migrate to the site of injury, and dead cells and pathogens are removed [155,156]. The proliferative phase involves fibroblast activation, extracellular matrix deposition, angiogenesis, and re-epithelialization, regulated by growth factors including TGF-β, FGF, PDGF, and VEGF [157]. The final phase is tissue remodeling, during which the newly formed tissue matures and reorganizes. Type III collagen is gradually replaced by type I collagen, increasing the mechanical strength of the scar. This phase is driven by extracellular matrix remodeling processes, regulated by matrix metalloproteinases and their inhibitors, as well as the continued activity of fibroblasts and endothelial cells [158].

Under physiological conditions, the scarring process occurs in a regulated manner and leads to the formation of a normal scar. Dysregulation at any phase of this process—often influenced by genetic, immunological, or mechanical factors—can give rise to pathological scars: atrophic, hypertrophic, or keloid [159]. Atrophic scars arise mainly due to insufficient collagen production and increased degradation of extracellular matrix components, particularly mediated by matrix metalloproteinases. Pathophysiology includes reduced fibroblast proliferation, impaired growth factor signaling (TGF-β1, FGF, PDGF), and reduced angiogenesis [160]. Hypertrophic scars are confined within the wound and are characterized by excessive deposition of the extracellular matrix, particularly type I collagen. The key perturbation at the molecular level is the over-activation of the TGF-β/Smad signaling pathway, which leads to a sustained stimulation of myofibroblasts—the cells responsible for the production of collagen fibers, which begin to produce smooth muscle cell-type protein (α-SMA) [161]. Recent findings have shown that downregulation of the CFTR ion channel affects fibroblast function, leading to decreased synthesis of type III collagen and an increased type I to type III collagen ratio, which promotes the formation of hypertrophic scars. CFTR dysfunction may also induce endoplasmic reticulum stress, which contributes to fibroblast activation and proliferation, as well as the development of fibrosis [162]. Keloids, in contrast to hypertrophic scars, extend beyond the boundaries of the original injury and do not tend to regress spontaneously. Their development is driven by severe dysregulation of apoptosis and persistent fibroblast activation. Keloid fibroblasts exhibit low expression of p53 and overexpression of Np63, which acts antagonistically to p53 by blocking apoptotic pathways. This promotes uncontrolled fibroblast proliferation and excessive collagen production. Keloid tissue also demonstrates sustained activation of pro-inflammatory cytokines (TNF-α, IL-6, IL-17) and hyperactivation of the TGF-β/Smad signaling pathway, further enhancing extracellular matrix deposition. Additionally, the balance between matrix metalloproteinases and their inhibitors is disrupted, leading to impaired extracellular matrix degradation [163,164,165]. Molecular mechanisms and therapeutic targets in different types of scars are summarized in Table 3.

### 5.2. Molecular Targets in Interventions for Atopic Dermatitis

Atopic dermatitis is a chronic, relapsing inflammatory dermatosis, clinically characterized by intense pruritus, xerosis, and recurrent eczematous lesions. The pathophysiology of atopic dermatitis is multifactorial, involving a complex interplay between immune dysregulation and epidermal barrier impairment. A critical factor contributing to barrier dysfunction is the presence of mutations in the FLG gene, which encodes filaggrin, a key structural protein responsible for the aggregation of keratin filaments and maintenance of skin hydration and integrity. Loss of function mutations in FLG compromise epidermal cohesion, resulting in increased transepidermal water loss and enhanced percutaneous penetration of allergens and irritants. The excessive presence of Staphylococcus aureus on the skin intensifies inflammation by releasing superantigens that strongly activate the immune system and disrupt epidermal balance [168]. This pathogen further damages the skin barrier, leading to increased inflammation and intensified itching. The colonization of atopic skin by *S. aureus* is facilitated by a disease-specific deficiency of antimicrobial peptides and impaired function of toll-like receptors, which are essential components of the innate immune response [169]. Emerging evidence underscores the pivotal role of the cutaneous microbiota in modulating immune responses and maintaining the functional integrity of the epidermal barrier, suggesting a dynamic and bidirectional relationship between microbial dysbiosis and the clinical course of atopic dermatitis [170]. Inflammatory pathways are central to the immunopathogenesis of atopic dermatitis. Cytokines such as tumor necrosis factor-alpha (TNF-α), interleukin IL-4, IL-13, and IL-31 have been identified as key molecular mediators in disease pathogenesis and symptomatology. TNF-α, secreted by monocytes, macrophages, and keratinocytes, facilitates the production of reactive oxygen species and pro-inflammatory cytokines, thereby amplifying the inflammatory environment within the skin. IL-4 and IL-13 are central to the type 2 immune response, promoting intercellular edema, impairing barrier function, and enhancing susceptibility to microbial colonization, particularly by S. aureus. IL-31, predominantly produced by activated Th2 cells, induces the expression of pro-inflammatory mediators and chemokines in keratinocytes. Furthermore, the upregulation of the IL-31 receptor alpha on peripheral sensory neurons is thought to contribute significantly to the pathogenesis of pruritus, a cardinal clinical feature of atopic dermatitis [169,171].

Daily skin care for patients with atopic dermatitis should be based on the systematic use of emollients, which aim to rebuild the damaged epidermal barrier. Patients with atopic dermatitis have a deficiency of lipids in the stratum corneum and reduced levels of natural moisturizing factor, which promotes excessive water loss through the epidermis. Emollients contain substances that rebuild the lipid barrier (e.g., ceramides, cholesterol, free fatty acids), act as occlusives such as petroleum jelly, paraffin, or lanolin, or bind water in the stratum corneum (glycerin, alpha hydroxyl acids and sorbitol) [172]. Active substances with multidirectional effects also play an important role in the daily care of skin with atopic dermatitis. Among them are cinnamic acid derivatives (including ferulic, caffeic, rosmarinic, and p-coumaric acids), which, thanks to their antioxidant, anti-inflammatory, and antibacterial properties, can support the treatment of atopic dermatitis [173].

#### Local Mediators

In atopic dermatitis, persistent activation of inflammatory pathways contributes to impaired epidermal function, disruption of the skin barrier, and the development of erythema and pruritus. Therefore, the ability of cinnamic acid derivatives to modulate various components of the inflammatory response may be relevant to the care of atopic skin.

Ferulic acid modulates the NF-κB pathway and reduces the production of pro-inflammatory mediators, such as TNF-α, IL-6, and nitric oxide, while also affecting the activity of COX-2 and iNOS [174,175]. These effects may help limit processes underlying persistent inflammation and impaired epidermal homeostasis. Caffeic acid influences the inflammatory response by modulating NF-κB and reducing the activity or expression of COX-2 and IL-8 [176]. These properties may be relevant to the care of atopic skin, where excessive production of pro-inflammatory mediators contributes to the persistence of inflammation and impaired skin barrier function. Chlorogenic acid affects several mechanisms associated with cutaneous inflammation. Its activity has been linked to attenuation of NF-κB signaling and reduced expression of TNF-α, IL-1β, IL-6, IL-8, TLR-2, and TLR-4 [177]. These properties suggest its potential usefulness in cosmetic formulations intended for the care of atopic skin. Rosmarinic acid affects several mechanisms involved in cutaneous inflammation. Its effects on NF-κB and MMP-9 may help attenuate inflammatory processes, while modulation of the MRGPRX2/PLCγ1 pathway may be relevant to pruritus [178,179]. Given the close relationship between pruritus and inflammation in atopic dermatitis, this activity may be relevant to the care of atopic skin.

Overall, these properties indicate the potential of these compounds as multifunctional cosmetic ingredients supporting the care of atopic skin, particularly by helping to alleviate inflammation and disturbances in epidermal homeostasis.

### 5.3. Molecular Targets in Hair Care

Hair growth is a highly regulated biological process governed by the cyclical activity of hair follicles, which undergo successive phases of growth (anagen), involution (catagen), and rest (telogen). Both hair follicle morphogenesis and cycle progression depend on the coordinated action of multiple signaling pathways. Dysregulation of these molecular networks can alter follicular homeostasis, affect hair shaft production, and promote conditions associated with hair thinning and hair loss [180,181].

#### Wnt/β-Catenin Signaling

Among the molecular pathways involved in hair follicle biology, Wnt/β-catenin signaling is considered a key regulator of follicular development and hair cycle progression. In particular, it plays a central role in the transition of hair follicles from the resting (telogen) phase to the active growth (anagen) phase. Moreover, this pathway is involved in virtually all stages of hair follicle development and plays a critical role in determining cell fate during follicle morphogenesis and regeneration [180,182]. Activation of this pathway stabilizes β-catenin, which subsequently translocates to the nucleus and induces the expression of genes responsible for stem cell activation, proliferation and follicular regeneration. As a result, Wnt/β-catenin signaling promotes hair follicle growth and regeneration, making it a promising molecular target for cosmetic ingredients aimed at supporting hair growth and maintaining follicular homeostasis [183,184]. Current evidence indicates that suppression of Wnt/β-catenin signaling by dihydrotestosterone represents a major mechanism contributing to the development of androgenetic alopecia. Restoration of Wnt/β-catenin activity may reverse these effects and improve the hair-inductive properties of DPCs, highlighting this pathway as a promising molecular target for the management of androgenetic alopecia [185].

Several cosmetic ingredients have been reported to modulate the Wnt/β-catenin pathway, including botanical extracts, peptides, and bioactive compounds capable of stimulating follicular stem cell activity and promoting hair growth. A number of plant-derived compounds targeting this pathway have been described, including loliolide, tocotrienol, 3,4,5-tri-O-caeoylquinic acid and sinapic acid, baicalin and silibnin, all of which have demonstrated the ability to enhance Wnt/β-catenin signaling and stimulate hair follicle activity in experimental models [184].

In 2023, Tang et al. [186] investigated exosomes isolated from adipose-derived stem cells (ADSC-Exos) and their effects on hair growth under androgenetic alopecia-like conditions. The study employed complementary in vitro, ex vivo, and in vivo models, including human dermal papilla cells exposed to dihydrotestosterone (DHT), cultured human hair follicles, and DHT-treated C57BL/6 mice. ADSC-derived exosomes promoted dermal papilla cell proliferation and migration, enhanced hair follicle growth, and prolonged anagen-associated activity. Importantly, ADSC-Exos partially reversed DHT-induced suppression of Wnt/β-catenin signaling, as evidenced by increased pGSK-3β and β-catenin levels, ultimately supporting hair follicle regeneration. In the animal model, treatment with ADSC-Exos stimulated the transition of hair follicles from telogen to anagen and promoted hair regrowth, demonstrating effects that were comparable or even superior to those observed for minoxidil [186].

Particularly noteworthy is PTD-DBM, a peptide specifically designed to disrupt the interaction between CXXC5 and Disheveled (Dvl), a central component of the Wnt signaling cascade. CXXC5 acts as a negative regulator of the Wnt pathway and was found to be highly expressed in miniaturized hair follicles of balding scalp tissue, suggesting its involvement in the pathogenesis of hair loss. By preventing the inhibitory binding of CXXC5 to Dvl, PTD-DBM restores Wnt/β-catenin signaling and promotes hair follicle regeneration. In experiments using human hair follicle dermal papilla cells, PTD-DBM increased the expression of β-catenin, alkaline phosphatase (ALP), and proliferating cell nuclear antigen (PCNA), indicating enhanced follicular activity and cellular proliferation. Furthermore, topical application of PTD-DBM in mouse models accelerated hair regrowth and promoted the transition of hair follicles into the anagen phase. Importantly, the peptide also stimulated wound-induced hair follicle neogenesis, a process associated with the formation of new hair follicles. These findings highlight the considerable regenerative potential of targeted CXXC5 inhibition and further support Wnt/β-catenin signaling as a promising molecular target for hair growth-promoting cosmetic formulations. Furthermore, PTD-DBM maintained its stimulatory effects in dermal papilla cells even in the presence of dihydrotestosterone (DHT), suggesting its potential relevance for conditions associated with androgen-driven hair loss [187].

Collectively, these findings demonstrate that modulation of Wnt/β-catenin signaling represents a promising strategy for the development of next-generation cosmetic products aimed at promoting hair growth and maintaining follicular homeostasis.

### 5.4. Molecular Targets in Nail Care

Nail growth and quality depend primarily on the activity of nail matrix keratinocytes, which are responsible for the continuous production of the nail plate. The nail plate is a highly keratinized structure composed of tightly packed keratinocytes and consists predominantly of hard keratins, complemented by smaller amounts of epithelial keratins. Its mechanical properties are determined by keratin organization, high sulfur content, and extensive protein cross-linking, particularly through cystine-derived disulfide bonds. Consequently, molecular processes regulating keratinocyte proliferation, differentiation, keratinization, and keratin fiber stabilization have emerged as potential targets for cosmetic ingredients designed to improve nail strength, resilience, and appearance [188,189].

#### Keratin Expression

Hard keratins account for approximately 80–90% of total nail keratin and are primarily expressed within the nail matrix, the region responsible for nail plate formation. These proteins are considered characteristic differentiation markers of the nail unit. Furthermore, experimental studies have demonstrated that hard keratin expression can be regulated through epithelial–mesenchymal interactions involving nail matrix fibroblasts, highlighting the importance of molecular mechanisms controlling keratinocyte differentiation and keratin expression as potential targets for improving nail strength and quality [190,191]. Because the mechanical properties of the nail plate largely depend on keratin content and organization, modulation of keratinization processes has attracted considerable interest in the development of nail cosmetic formulations intended to reduce brittleness and improve nail resilience.

One example of targeting keratinization- and keratinocyte differentiation-associated pathways is described in a patent by Bondil et al. [192]. The authors investigated a supercritical CO_2_ extract of *Apium graveolens* seeds enriched in alkyl-phthalides, principally sedanenolide, sedanolide, and 3-n-butylphthalide. The extract contained at least 50% alkyl-phthalides by weight and was specifically developed for the cosmetic treatment of soft, brittle, and split nails, and nails with longitudinal ridges. In vitro studies indicated that the extract modulated the Wnt/β-catenin pathway and promoted markers associated with keratinocyte differentiation and cornification. In particular, treatment increased WNT10A expression by 49% and reduced the expression of DKK1, a physiological inhibitor of Wnt/β-catenin signaling, by 45%. The extract also increased the expression of several differentiation- and cornification-associated markers, including involucrin, loricrin, filaggrin, and trichohyalin. In addition, increased expression of the keratins K6, K16, and K17 was observed in nail samples collected from participants following the treatment period, with increases of approximately 41%, 93%, and 53%, respectively. The biological findings were accompanied by results from a 4-month in vivo study. Treatment with the *A. graveolens* extract increased nail matrix density and nail growth rate and was associated with an increase in nail plate thickness. A reduction in transonychial water loss (TOWL) was also observed, together with reduced nail dryness and longitudinal ridging. These findings, together with the observed modulation of keratinocyte differentiation and keratinization-associated markers, suggest that alkyl-phthalide-rich *A. graveolens* extract may support nail structure and quality through effects on keratinocyte differentiation, cornification, and nail matrix function [192].

## 6. Materials and Methods

A comprehensive literature search was carried out on PubMed, Scopus, Web of Science, and Google Scholar, covering the period from June 2025 to August 2026. The initial search focused on molecular targets relevant to cosmetics and cosmetology, using combinations of the following terms: “molecular targets cosmetic”, “molecular targets skin”, “molecular targets hair care”, and “molecular targets cosmetology”. Additional targeted searches were subsequently undertaken for individual molecular pathways and targets associated with skin aging, telomerase, histone deacetylases, matrix metalloproteinases, antioxidant enzymes, the CD44 receptor, tyrosinase, MITF, melanocortin receptors, scar formation, atopic dermatitis, hair care, and nail care.

No publication-date limits were imposed, allowing both seminal studies and more recent evidence to be considered. Eligible publications were English-language articles addressing the cosmetic, dermatological, or mechanistic relevance of molecular targets in skin and hair, including original research, clinical investigations, and peer-reviewed review articles. Studies dealing exclusively with systemic or pharmacological applications and lacking a direct connection to cosmetic use were excluded. The potential application of individual compounds in cosmetic products was additionally assessed using the CosIng database, while their regulatory status and restrictions were checked against the relevant cosmetic legislation.

During the preparation of this work the authors used ChatGPT 5.6 for English language grammar editing and refinement. After using this tool, the authors reviewed and edited the content as needed and take full responsibility for the content of the publication.

## 7. Conclusions

Recent advances in molecular biology and cosmetology have significantly reshaped the approach to skin care and esthetic interventions. Rather than addressing only visible symptoms, modern cosmetology increasingly focuses on molecular-level mechanisms underlying processes such as aging, pigmentation, inflammation, skin regeneration, and good hair and nail condition. Key biological macromolecules, including telomerase, histone deacetylases, matrix metalloproteinases, antioxidant enzymes, CD44 adhesion receptor, tyrosinase, microphthalmia-associated transcription factor, melanocortin receptors, and Wnt/β-catenin signaling have emerged as strategic molecular targets in the development of innovative cosmetic ingredients.

Among these, the role of telomerase and telomere maintenance has become a cornerstone in anti-aging interventions, with evidence suggesting that certain plant-derived compounds and peptides may modulate telomerase activity and improve skin regenerative capacity. Epigenetic regulators, particularly histone deacetylases such as sirtuins, represent promising targets for modulating gene expression related to longevity and cellular repair. Compounds affecting these pathways hold potential for use in “genocosmetics”—a new generation of personalized skin care products that aim to modulate gene activity without crossing into therapeutic claims. However, although several cosmetic ingredients show encouraging effects, in many cases, robust scientific evidence confirming their cellular mechanisms of action remains limited.

In pigmentation control, the shift from traditional assays based on mushroom tyrosinase to human enzyme models has facilitated the discovery of highly selective melanogenesis inhibitors, such as thiamidol. These findings offer hope for safer and more effective depigmenting agents. Furthermore, the modulation of melanocortin receptors and tissue mediators provides new directions for both skin tone regulation and management of inflammatory skin conditions like atopic dermatitis.

**Figure 4:**
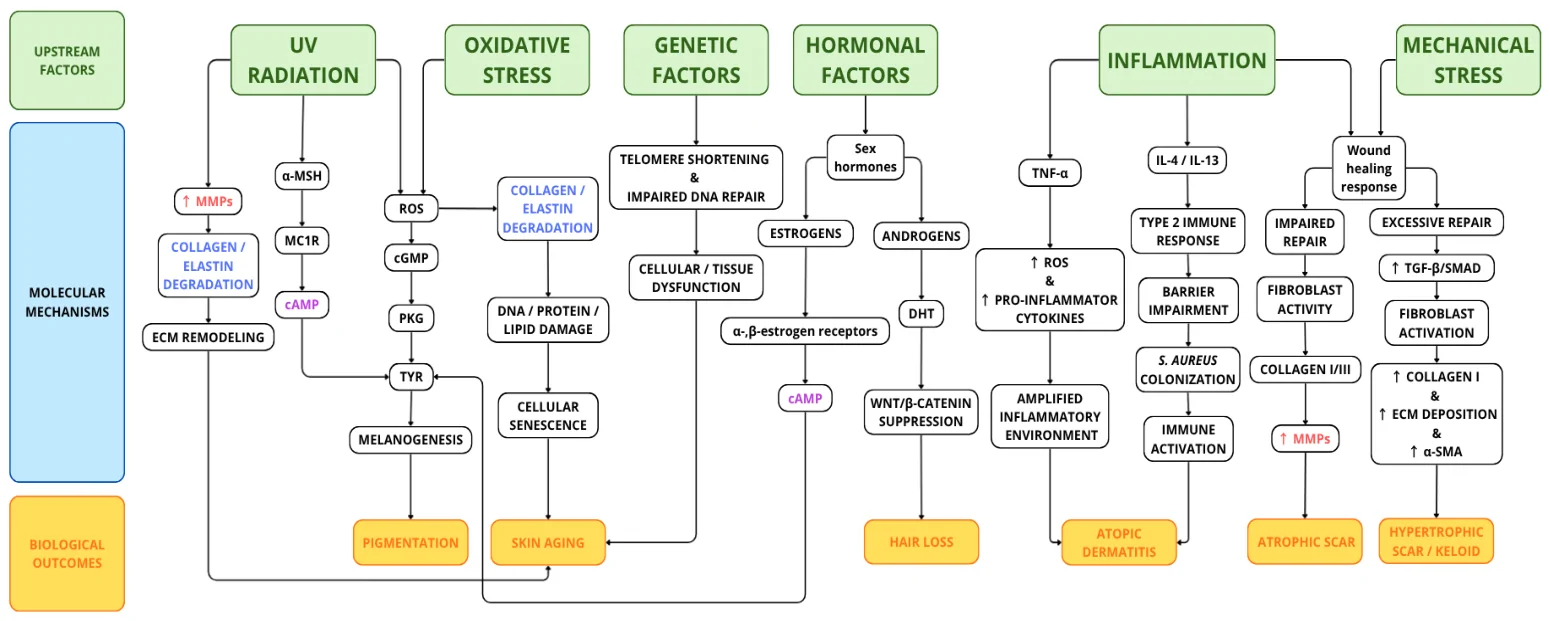
Schematic representation of relationships between internal and external factors affecting key biological outcomes in modern cosmetology (α-MSH—alpha-melanocyte-stimulating hormone, α-SMA—alpha-smooth muscle actin, cAMP—cyclic adenosine monophosphate, cGMP—cyclic guanosine monophosphate, DHT—dihydrotestosterone, ECM—extracellular matrix, MC1R—melanocortin 1 receptor, MMPs—matrix metalloproteinases, PKG—protein kinase G, ROS—reactive oxygen species, TYR—tyrosinase, UV—ultraviolet, Wnt—Wingless-related integration site), ↑—increase.

The following diagram (Figure 4) illustrates the relationships between internal and external factors and key molecular pathways involved in pigmentation, skin aging, hair loss, atopic dermatitis, and pathological scar formation. The diagram highlights shared molecular regulators, illustrating the interconnected nature of these processes.

While significant progress has been made, the cosmetic application of molecular-targeted compounds requires further investigation, particularly with respect to safety, efficacy, long-term outcomes, and regulatory compliance. Continued development of non-invasive diagnostic tools, such as high-resolution skin imaging, dermoscopy, and instrumental assessment of skin hydration, elasticity, pigmentation, and barrier function; advanced in vitro testing platforms, including reconstructed human skin models, three-dimensional skin equivalents, and skin organoids; and innovative delivery systems, such as lipid-based nanoparticles, liposomes, and other targeted delivery technologies, will be essential to support the development and evaluation of molecular-targeted cosmetic products. These approaches may facilitate more precise characterization of skin condition, improved assessment of cosmetic efficacy and safety, and enhanced delivery of bioactive ingredients to relevant skin compartments.

Overall, the strategic exploration of biological macromolecules offers a transformative framework for future cosmetological innovations, combining scientific rigor with the growing demand for personalized, evidence-based skin care solutions. Future research should prioritize multi-target approaches, recognizing that skin aging results from the interplay of multiple interconnected molecular pathways rather than a single mechanism. Further investigation of the combined modulation of telomere maintenance, sirtuin-mediated epigenetic regulation, MMP activity, and antioxidant defense may provide valuable insights into the development of more effective cosmetic strategies. Personalized target discovery supported by omics technologies may further contribute to the identification of molecular pathways associated with individual skin aging profiles and facilitate the development of more personalized cosmetic formulations. The validation of candidate ingredients will increasingly benefit from advanced three-dimensional skin models and organoids, which more closely reproduce the architecture and biological characteristics of human skin than conventional two-dimensional cultures. Overall, the molecular targets discussed in this review provide a promising framework for the future development of evidence-based cosmetic products aimed at supporting skin health and addressing the visible signs of skin aging. Realizing this potential will require continued multidisciplinary collaboration across molecular biology, cosmetology, computational science, and regulatory research.

## Figures and Tables

**Table 2:** Main differences between human tyrosinase and mushroom tyrosinase (according to [109,112,113,114,115]).

Feature	Human Tyrosinase	Mushroom Tyrosinase
Cell localization	Melanosome membrane	Melanosome cytosol
Form	Monomeric, glycosylated	Oligomeric (tetramer), soluble
Molecular weight	75 kDa	120 kDa
Specific domains	Unique cysteine-rich subdomain located at the cytosol of melanosomes (the EGF domain), a transmembrane hydrophobic domain, and seven asparagine glycosylation sites (N86, N111, N161, N230, N290, N337, N371)	Absent
Additional features at the active site	Hot spot residues H304, K306, R308, T343, T352, I368, S375 or S380	A covalent thioether bond between His85 and Cys83

**Table 3:** Molecular mechanisms and therapeutic targets in different types of scars.

Scar Type	Molecular Mechanisms	Therapeutic Targets
Atrophic	↓ Collagen I/III, ↓ TGF-β, ↓ FGF/PDGF [157]	Neocollagenesis stimulation, MMP inhibition [157]
Hypertrophic	↑ TGF-β/Smad [165,166] ↓ CFTR, altered collagen I/III ratio, ER stress [162]	TGF-β pathway inhibition [166], Collagen balance restoration, ER stress reduction [162]
Keloid	↑ CLC-3 [160], ↑ IL-17 [167]	Regulation of apoptosis [163,164,167]

↓—decrease, ↑—increase.

## Data Availability

No new data were created or analyzed in this study. Data sharing is not applicable to this article.
